# Conformational Change-Induced Repeat Domain Expansion Regulates Rap Phosphatase Quorum-Sensing Signal Receptors

**DOI:** 10.1371/journal.pbio.1001512

**Published:** 2013-03-19

**Authors:** Vijay Parashar, Philip D. Jeffrey, Matthew B. Neiditch

**Affiliations:** 1 Department of Microbiology and Molecular Genetics, UMDNJ–New Jersey Medical School, Newark, New Jersey, United States of America; 2 Department of Molecular Biology, Princeton University, Princeton, New Jersey, United States of America; HHMI, Massachusetts Institute of Technology, United States of America

## Abstract

The large family of Gram-positive quorum-sensing receptors known as the RNPP proteins consists of receptors homologous to the *R*ap, *N*prR, *P*lcR, and *P*rgX proteins that are regulated by imported oligopeptide autoinducers. Rap proteins are phosphatases and transcriptional anti-activators, and NprR, PlcR, and PrgX proteins are DNA binding transcription factors. Despite their obvious importance, the mechanistic basis of oligopeptide receptor regulation is largely unknown. Here, we report the X-ray crystal structure of the *Bacillus subtilis* quorum-sensing receptor RapJ in complex with the centrally important oligopeptide autoinducer competence and sporulation factor (CSF, also termed PhrC), a member of the Phr family of quorum-sensing signals. Furthermore, we present the crystal structure of RapI. Comparison of the RapJ-PhrC, RapI, RapH-Spo0F, and RapF-ComA_C_ crystal structures reveals the mechanistic basis of Phr activity. More specifically, when complexed with target proteins, Rap proteins consist of a C-terminal tetratricopeptide repeat (TPR) domain connected by a flexible helix-containing linker to an N-terminal 3-helix bundle. In the absence of a target protein or regulatory peptide, the Rap protein 3-helix bundle adopts different conformations. However, in the peptide-bound conformation, the Rap protein N-terminal 3-helix bundle and linker undergo a radical conformational change, form TPR-like folds, and merge with the existing C-terminal TPR domain. To our knowledge, this is the first example of conformational change-induced repeat domain expansion. Furthermore, upon Phr binding, the entire Rap protein is compressed along the TPR superhelical axis, generating new intramolecular contacts that lock the Rap protein in an inactive state. The fact that Rap proteins are conformationally flexible is surprising considering that it is accepted dogma that TPR proteins do not undergo large conformational changes. Repeat proteins are widely used as scaffolds for the development of designed affinity reagents, and we propose that Rap proteins could be used as scaffolds for engineering novel ligand-switchable affinity reagents.

## Abstract 2

Structure-function studies reveal hojavascript:popupCustomFlags(‘pbiology’,%2013052,%20‘Submission’)w a family of bacterial cell-cell signaling peptides function mechanistically to regulate their cytoplasmic target receptors.

## Author Summary

The bacterial cell–cell communication process known as quorum sensing regulates important social behaviors including antibiotic production, motility, virulence, biofilm formation, sporulation, bioluminescence, and genetic competence. Gram-positive bacteria secrete oligopeptide quorum-sensing signals that bind to membrane-bound and cytosolic receptors. How oligopeptide quorum-sensing signals regulate the activity of their target receptors was previously largely unknown. Here we show that proteins belonging to the family of bacterial quorum-sensing receptors known as the Rap phosphatases undergo a remarkable regulatory conformational change upon binding oligopeptide signals. More specifically, in the absence of the oligopeptide signal, Rap proteins consist of two distinct domains: an N-terminal domain consisting of a three-helix bundle, and a superhelical C-terminal domain comprising an array of seven similar helix-turn-helix repeats. A flexible helix-containing linker region connects these domains. In complex with the regulatory oligopeptide, however, the Rap protein domains and linker region rearrange, merging to form a single continuous superhelical structure consisting of nine helix-turn-helix repeats. To our knowledge, this represents the first example of conformational change-induced repeat domain expansion. The structure-function studies presented here set the stage for the rational development of antimicrobial peptides and peptide-mimetics capable of targeting cell–cell signaling mediated by Rap proteins and similar bacterial receptors.

## Introduction

Quorum sensing is a bacterial cell–cell communication process mediated by secreted signaling molecules. At low cell density, the concentration of the quorum-sensing signals is negligible and bacteria act as individuals. At high cell density, the concentration of the signals is sufficient to coordinate bacterial social behaviors including sporulation, virulence factor expression, motility, biofilm formation, bioluminescence, antibiotic production, and genetic competence [1]. Typically, acylated homoserine lactones are used as quorum-sensing signals by Gram-negative bacteria, whereas oligopeptides are used by Gram-positive bacteria. Despite their obvious importance, the mechanistic basis of oligopeptide receptor regulation in Gram-positive species is largely unknown.

**Figure 1:**
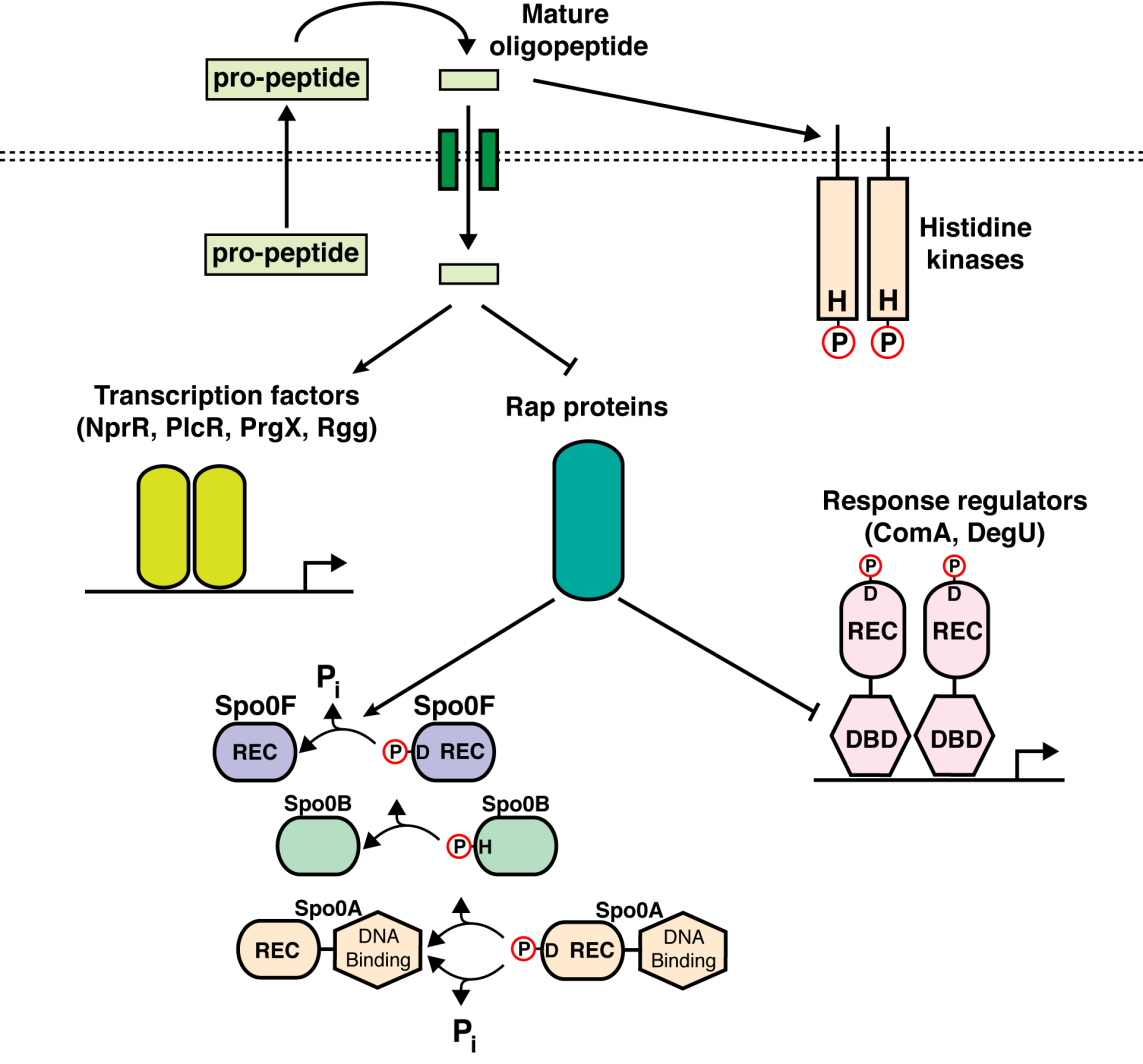
Secreted oligopeptide regulation of bacterial quorum-sensing receptors. Secreted oligopeptide signals are synthesized as immature pro-peptides, which are then processed and secreted. The mature oligopeptides bind to and regulate histidine kinases belonging to two-component or phosphorelay signal transduction systems, or the oligopeptides otherwise bind to oligopeptide permeases, which import the oligopeptides into the cell. Here the oligopeptides bind to and regulate (activate or, in some cases, inhibit) the activity of DNA binding transcription factors, such as *Streptococcus* Rgg, *Bacillus* PlcR and NprR, or *Enterococcus* PrgX. Alternatively, the oligopeptides bind to and regulate the Bacillus Rap proteins. The Rap proteins function as phosphatases targeting intermediate response regulators such as Spo0F belonging to the sporulation phosphorelay pathway that activates (phosphorylates) the master response regulator Spo0A, or the Rap proteins function as transcriptional anti-activators targeting response regulator transcription factors such as ComA and DegU. H, histidine; D, aspartate; P, phosphoryl group; P_i_, inorganic phosphate; REC, receiver domain; DBD, DNA Binding Domain.

Secreted oligopeptide signals are commonly synthesized as immature pro-peptides (Figure 1) [2]. The genes encoding the pro-peptides are usually encoded immediately upstream or downstream of their cognate receptor genes, forming receptor–pro-peptide gene cassettes. The immature pro-peptides are secreted from the cell and subsequently undergo proteolytic maturation [3]. The mature oligopeptides bind to and regulate transmembrane receptors such as histidine kinases, or alternatively, the mature oligopeptides are imported into the cell by oligopeptide permeases [4]–[7]. Inside the cell, the oligopeptides bind to and regulate target receptors [7]–[11]. These cytoplasmic receptors include (1) members of the RNPP protein family, consisting of receptors homologous to the *R*ap, *N*prR, *P*lcR, or *P*rgX proteins, which are widespread in Firmicutes (e.g., *Bacillus* and *Enterococcus* species) [9],[12]–[14], and (2) the Rgg proteins, which are ubiquitous in *Streptococcus* and many other low G+C Gram-positive species [15]. NprR, PlcR, PrgX, and Rgg proteins are DNA binding transcription factors. In contrast, as described below, Rap proteins have diverse catalytic and noncatalytic activities, and Rap proteins are not DNA binding transcription factors.

Rap proteins and their inhibitory oligopeptides, called Phr peptides, have been most extensively studied in *B. subtilis*, which encodes 11 Rap proteins on its chromosome and another five on plasmids [16]–[18]. The founding members of the Rap family, RapA and RapB, were shown to be response regulator aspartate phosphatases (Rap) [19]. Subsequently, RapE, RapH, and RapJ were demonstrated to be response regulator aspartate phosphatases [20]–[22], and RapC, RapF, RapG, and RapH were revealed to be transcriptional anti-activator proteins that inhibit the binding of the response regulators ComA or DegU to DNA promoters [20],[23]–[25].

Genes encoding pro-Phr polypeptides overlap with the 3′ end of *rap* genes, forming *rap-phr* gene cassettes. Mature Phr peptides are imported into the cell where each oligopeptide inhibits its cognate Rap protein (e.g., PhrA inhibits RapA, and PhrC inhibits RapC) and in some cases a non-cognate Rap protein (e.g., PhrC inhibits RapB, which is not encoded in a cassette with a cognate *phr* gene) [10],[26],[27]. Like *rapB*, *rapJ* is not encoded in an operon with a *phr* gene, and here we show that the centrally important Phr peptide PhrC, which is also commonly referred to as competence and sporulation factor [6],[27], binds to RapJ and inhibits its phosphatase activity.

While it is well-established that Phr peptides bind to Rap proteins and inhibit their activity, how Phr peptides regulate Rap proteins remained unknown. Here we report two X-ray crystal structures containing Rap proteins: the structures of a Rap-Phr complex, RapJ-PhrC, and a Rap protein, RapI, alone. These structures and supporting in vivo and in vitro studies, together with the previously determined structure of (1) RapH complexed with the intermediate response regulator Spo0F [22] and (2) the structure of RapF complexed with the DNA binding domain of the transcription factor ComA (ComA_C_) [28], reveal the mechanistic basis of Rap protein regulation by Phr peptides.

Interestingly, our structure-function analysis shows that Rap proteins exist in radically different conformations in the target-bound, Phr peptide-bound, and ligand-free conformations. The fact that Rap proteins undergo dramatic conformational changes is particularly surprising because Rap proteins are tetratricopeptide repeat (TPR) proteins, which were believed to be rigid frameworks that do not undergo large conformational changes. Repeat proteins are widely used as scaffolds for the development of designed affinity reagents, and our results suggest that Rap proteins could be used as scaffolds for engineering or evolving novel ligand-switchable TPR-based affinity reagents. In addition, we note that the studies presented here set the stage for the rational development of antimicrobial peptides and peptide-mimetics targeting Rap-mediated cell–cell signaling.

## Results

### X-Ray Crystal Structure of the RapJ-PhrC Complex

**Figure 2:**
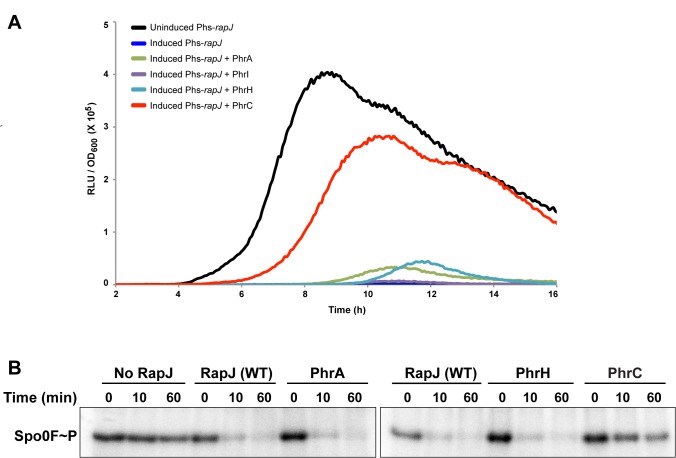
Activity of Phr peptides on RapJ phosphatase activity in vivo and in vitro. (A) RapJ activity was measured as a function of P*spoIIG*::*luc* expression in strain VP068. Each curve is representative of at least three independent experiments performed in duplicate. RapJ overexpression was controlled by the IPTG-inducible promoter P_hyperspank_ (Phs), and the oligopeptides were used at 530 µM. RLU, Relative Luminescence Units. (B) The ability of RapJ to dephosphorylate Spo0F∼P in the presence and absence of Phr peptides was compared. RapJ was used at 6.5 µM, and the Phr oligopeptides were used at 310 µM. The gels are representative of experiments repeated at least three times.

Our primary goal was to determine the mechanistic basis of Rap protein regulation by Phr peptides using a combination of X-ray crystallographic, biochemical, and genetic approaches. Despite extensive efforts, we had only limited success crystallizing previously identified Rap-Phr pairs, and we therefore sought to identify new Rap-Phr pairs to target for crystallization. We previously showed in vitro that RapJ dephosphorylates Spo0F [22], an intermediate response regulator in the *B. subtilis* sporulation phosphorelay pathway [29]; however, because *rapJ* is not encoded in an operon with a *phr* gene, whether a RapJ regulatory peptide existed was unknown. To determine whether previously identified Phr peptides inhibit RapJ Spo0F phosphatase activity, we measured the ability of synthetic Phr peptides to inhibit RapJ dephosphorylation of Spo0F in vivo as a function of Spo0A activation using a luciferase gene under the control of the Spo0A-driven promoter P*spoIIG* (Figure 2A). As expected, overexpressing RapJ completely repressed P*spoIIG* activation (Figures 2A and S1A), and deleting *rapJ* resulted in elevated P*spoIIG* expression (Figure S1A). Surprisingly, however, PhrC restored P*spoIIG* expression to levels approaching that of the negative control—that is, when RapJ overexpression was not induced (Figures 2A and S1A). In contrast, other Phr peptides tested—e.g., PhrA (ARNQT) and PhrH (TDRNTT)—had little or essentially no effect on P*spoIIG* expression (Figure 2A and unpublished data).

To determine whether RapJ and PhrC interact directly, we incubated purified RapJ with synthetic PhrC and subjected the sample to size exclusion chromatography (SEC). Using MALDI-TOF and MALDI-TOF/TOF tandem mass spectrometry, we detected PhrC complexed with RapJ (Figure S2); control experiments confirmed that SEC separates the RapJ-PhrC complex from unbound PhrC (Figure S3).

To confirm that PhrC directly inhibits RapJ dephosphorylation of Spo0F, we phosphorylated Spo0F as previously described [22] and measured the ability of synthetic PhrC to inhibit RapJ phosphatase activity in vitro. Consistent with the above in vivo results, PhrC inhibited RapJ-accelerated dephosphorylation of Spo0F (Figure 2B). Other Phr peptides had no effect on RapJ Spo0F phosphatase activity (Figure 2B and unpublished data). Based on the above in vivo and in vitro analyses, we conclude that PhrC directly inhibits RapJ Spo0F phosphatase activity; however, we note PhrC has at least three targets (RapB, RapC, and RapJ) and the physiological importance of RapJ inhibition by PhrC is unknown [10],[27].

**Figure 3:**
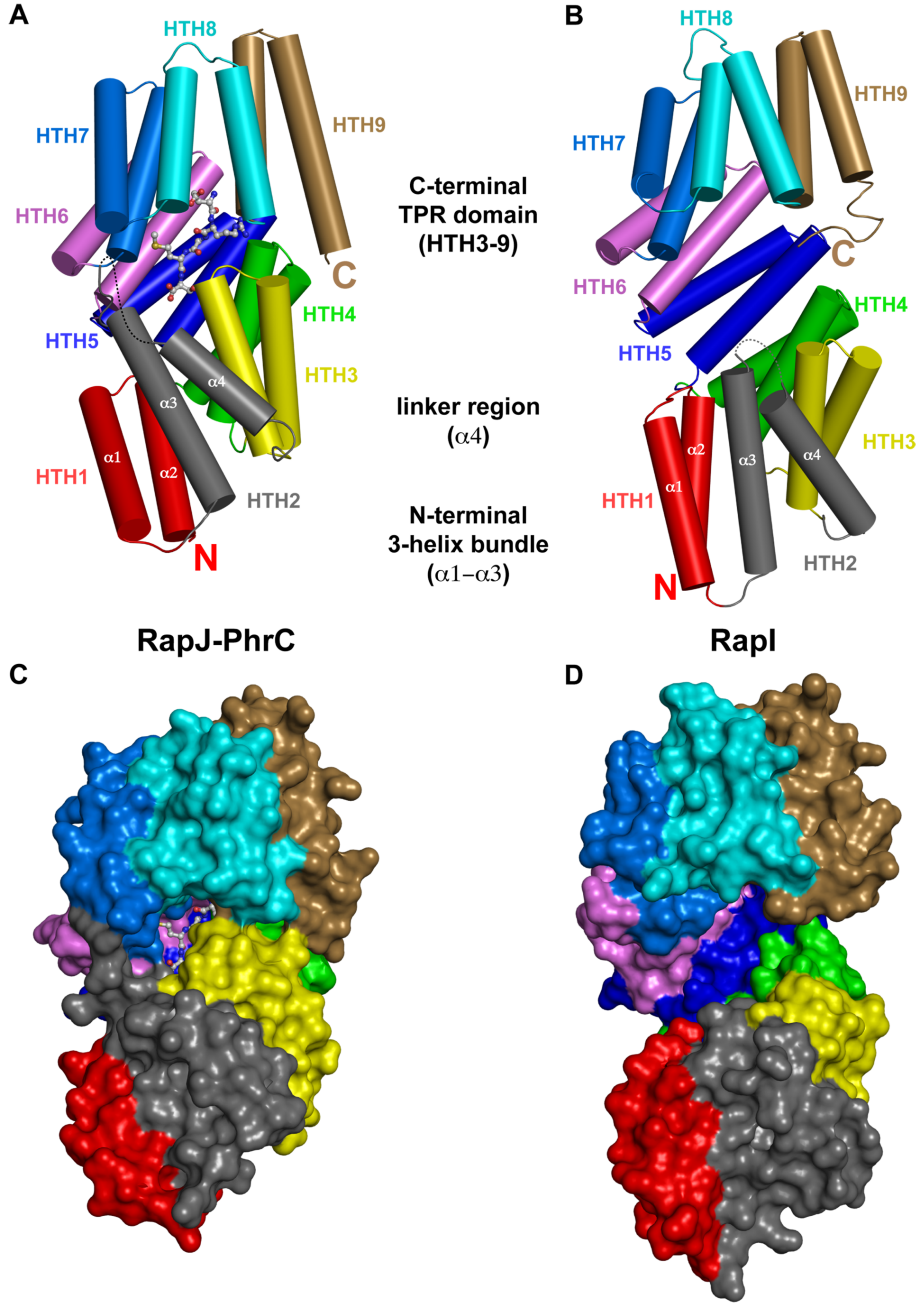
RapJ-PhrC and RapI X-ray crystal structures. (A) The X-ray crystal structure of RapJ (rainbow cartoon with α-helices depicted as cylinders) complexed with PhrC (ball and stick model). (B) The X-ray crystal structure of RapI alone (rainbow cartoon with α-helices depicted as cylinders). (C) The X-ray crystal structure of RapJ (rainbow surface) complexed with PhrC (ball and stick model). (D) The X-ray crystal structure of RapI alone (rainbow surface). Comparison of the RapI and RapJ-PhrC structures shows that the RapI TPR domain ligand-binding groove is open, while the RapJ-PhrC TPR ligand-binding groove is closed. HTH, helix-turn-helix; N, amino-terminus; C, carboxyl-terminus.

Following the discovery that RapJ and PhrC form a regulatory pair, we crystallized and determined the 2.16 Å resolution X-ray crystal structure of the RapJ-PhrC complex (Figure 3A). Straightforward approaches to obtain phases for the RapJ-PhrC structure by molecular replacement using our previously determined structures of Rap proteins were unsuccessful, suggesting that PhrC binding had induced a large conformational change in RapJ; therefore, we overexpressed and purified selenomethionyl derivatized RapJ and determined phases for the RapJ-PhrC complex using the single wavelength anomalous dispersion (SAD) method (Table S1). Readily interpretable electron density corresponding to each residue in the PhrC pentapeptide was observed, and PhrC was modeled only after the RapJ model was nearly complete.

It is also important to note that while there are two copies of RapJ-PhrC in the asymmetric unit, sedimentation equilibrium (SE) analytical ultracentrifugation (AUC) shows that RapJ is monomeric in solution in the presence and absence of PhrC (Figure S4). More specifically, the theoretical molecular weights of the RapJ and RapJ-PhrC monomers are 44.41 kD and 45.01 kD, respectively, and their molecular weight as determined by SE AUC are 46.0 kD and 47.5 kD, respectively (Figure S4A,B). Consistent with these results, we previously reported that RapF, RapH, and RapK are monomeric in solution as determined by SE AUC [28].

### X-Ray Crystal Structure of RapI

The conformation adopted by Rap proteins in the absence of Phr peptide or target protein was unknown. To reveal the structure of a Rap protein alone, we crystallized and determined the 2.44 Å resolution X-ray crystal structure of RapI. As detailed in the Materials and Methods section, we obtained phases for the RapI structure by starting with a core search model consisting of a RapJ domain highly homologous to RapI, and then iteratively rebuilding and enlarging search models using phenix.mr_rosetta [30],[31] and scoring the models based on the LLG in the Phaser [32] rotation function (Figure 3B and Table S1). While discussed extensively below, it is worthwhile to note here that there are two copies of RapI in the crystallographic asymmetric unit, and while one model is relatively complete (Figure 3B), there was in fact insufficient electron density to build helix-turn-helix (HTH) 1 and HTH2 in the second model (see Discussion and Materials and Methods).

### RapI Is a Spo0F Phosphatase

**Figure 4:**
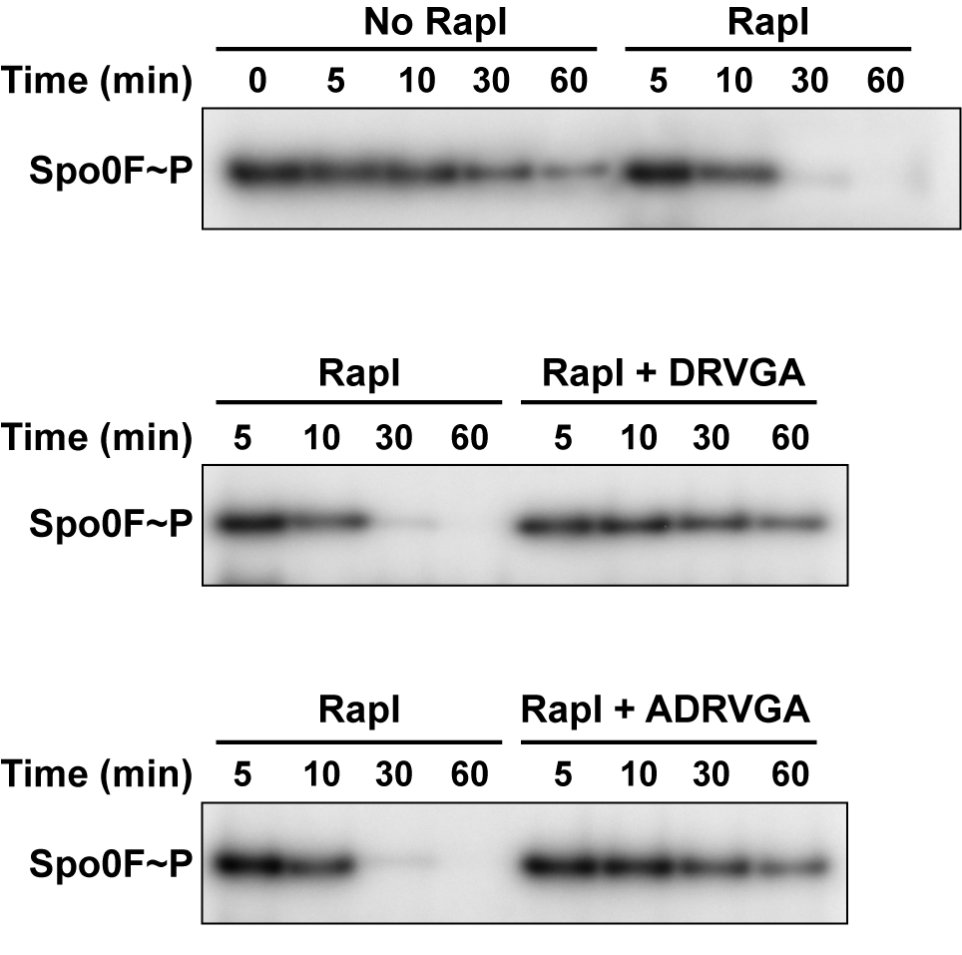
In vitro analysis of RapI phosphatase activity and its inhibition by PhrI peptides. RapI dephosphorylates Spo0F (top panel), and RapI Spo0F phosphatase activity is inhibited by the pentapeptide DRVGA (middle panel) and hexapeptide ADRVGA (bottom panel). RapI was used at 6.5 µM, and the Phr oligopeptides were used at 32.5 µM. The gels are representative of phosphatase assays repeated at least three times.

*B. subtilis* RapI was previously reported to stimulate the activity of the ImmA protease using an unknown mechanism, resulting in the expression, excision, and transfer of the conjugative transposon ICEBs1 [33]. However, following our structural analysis of RapI, we realized that it conserves 17 of 18 highly conserved residues in the Rap Spo0F interface (Table S2) [22], including the catalytic glutamine that inserts into the Spo0F active site. Therefore, we evaluated the ability of RapI to dephosphorylate Spo0F in vitro, and we found that RapI is indeed a Spo0F phosphatase (Figure 4). The fact that RapI activates ICEBs1 and dephosphorylates Spo0F suggests that RapI may be important for inhibiting sporulation during active ICEBs1 transposition. Furthermore, we determined that the pentapeptide DRVGA and hexapeptide ADRVGA, sequences derived from the pro-PhrI C-terminus, inhibit RapI phosphatase activity in vitro (Figure 4). We previously hypothesized that the hexapeptide form of PhrI would inhibit RapI and might also serve as the biologically important PhrI peptide [34].

### Rap Proteins Undergo Large Conformational Changes

**Figure 5:**
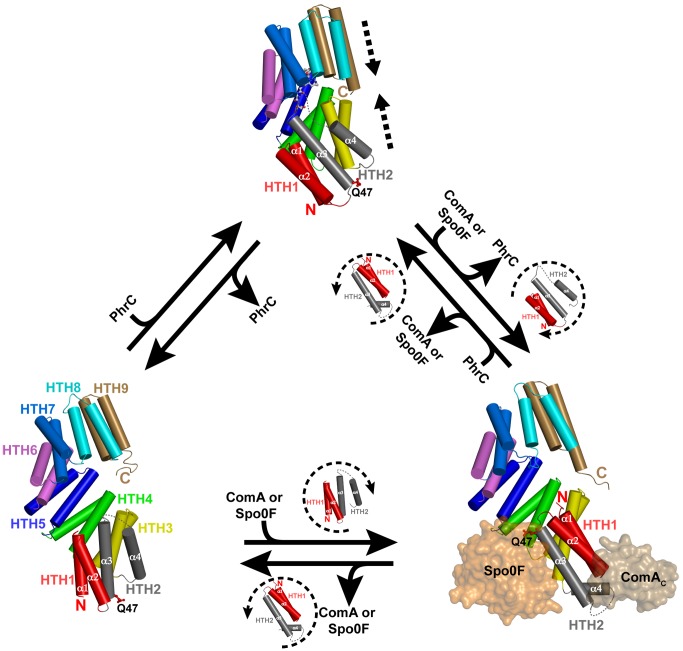
Rap protein conformations. The structures depicted show a Rap protein alone (bottom left, RapI), Rap-target protein complexes [bottom right, RapH-Spo0F (PDB ID 3Q15) and RapF-ComA_C_ (PDB ID 3ULQ)—for the sake of clarity RapF is omitted], and a Rap protein in complex with Phr peptide (top, RapJ-PhrC). The models surrounded by dashed arcs depict the movement of the Rap protein HTH1 and HTH2. In every panel, the sidechain of the catalytic glutamine corresponding to RapH Q47, RapJ Q47, and RapI Q53 is shown and labeled Q47. RapI alone (bottom left) is in an intermediate open conformation where HTH1 (red cylinders) and HTH2 (grey cylinders) extend the TPR domain (HTH3-HTH9) by two HTH repeats. RapH and RapF (bottom right) are in the fully open conformation, and they consist of an N-terminal 3-helix bundle (helices α1–α3), connected by the helix-α4 linker region, to a C-terminal TPR domain (HTH3-HTH9, colored as in the bottom left panel). RapJ-PhrC (top panel) is in the closed conformation. The direction of the Rap domain movements along the TPR domain superhelical axis are depicted by dashed arrows in the top panel.

Structural comparison of the Rap proteins in the previously determined structures of RapH-Spo0F and RapF-ComA_C_ with the structure of RapJ-PhrC revealed that Rap proteins undergo radical conformational changes (Figures 3A,B and 5, and Movie S1). We previously showed that Rap proteins are composed of two distinct domains when complexed with target proteins such as Spo0F or ComA (Figure 5) [22],[28]. These Rap domains are an N-terminal 3-helix bundle and a C-terminal TPR domain; a flexible helix-containing linker region connects the domains (Figure 5). Relative to its position in the RapH-Spo0F and RapF-ComA_C_ structures, the entire N-terminal 3-helix bundle and linker region have dramatically flipped and repacked against the N-terminal surface of the C-terminal TPR domain in the RapJ-PhrC structure (Figure 5). In fact, the RapJ 3-helix bundle (residues 1–70) and helix-containing linker region (residues 71–94) merge with the C-terminal TPR domain (residues 95–373) to form one extended TPR domain (residues 1–373) (Figures 3A,B, and 5). Each HTH consists of an A and B helix connected by a short loop. HTH1 is formed by helices α1 and α2 (the first two helices of the 3-helix bundle). HTH2 is formed by helix α3 (the third helix of the 3-helix bundle) and helix α4 (the linker region helix in the RapH-Spo0F and RapF-ComA_C_ structures). While HTH1 and HTH3–HTH7 conserve a number of the TPR motif signature residues, HTH2 forms a TPR-like fold but does not strictly conserve the TPR signature motif residues [35].

Similarly, comparing the structure of RapI with the structures of RapH-Spo0F and RapF-ComA_C_ revealed that the RapI 3-helix bundle and linker region have joined the TPR domain (Figure 5); however, it is important to note that RapI and RapJ are not in identical conformations (Figure 3A and 3B). The Rap protein HTH folds assemble into a right-handed superhelical structure (the TPR domain). TPR domains typically have a convex outer surface and a concave inner surface, commonly referred to as the ligand-binding groove. The RapI structure reflects this scenario, and the ligand-binding groove is in an open conformation (Figure 3B and 3D). In contrast, in the RapJ-PhrC structure, the ligand-binding groove is closed (Figure 3A and 3C). PhrC binding stabilizes a closed RapJ conformation that differs from the open RapI conformation due to a compression of RapJ along the TPR superhelical axis (Figures 3 and 5).

### Functional Analysis of the RapJ-PhrC Interface In Vivo

**Figure 6:**
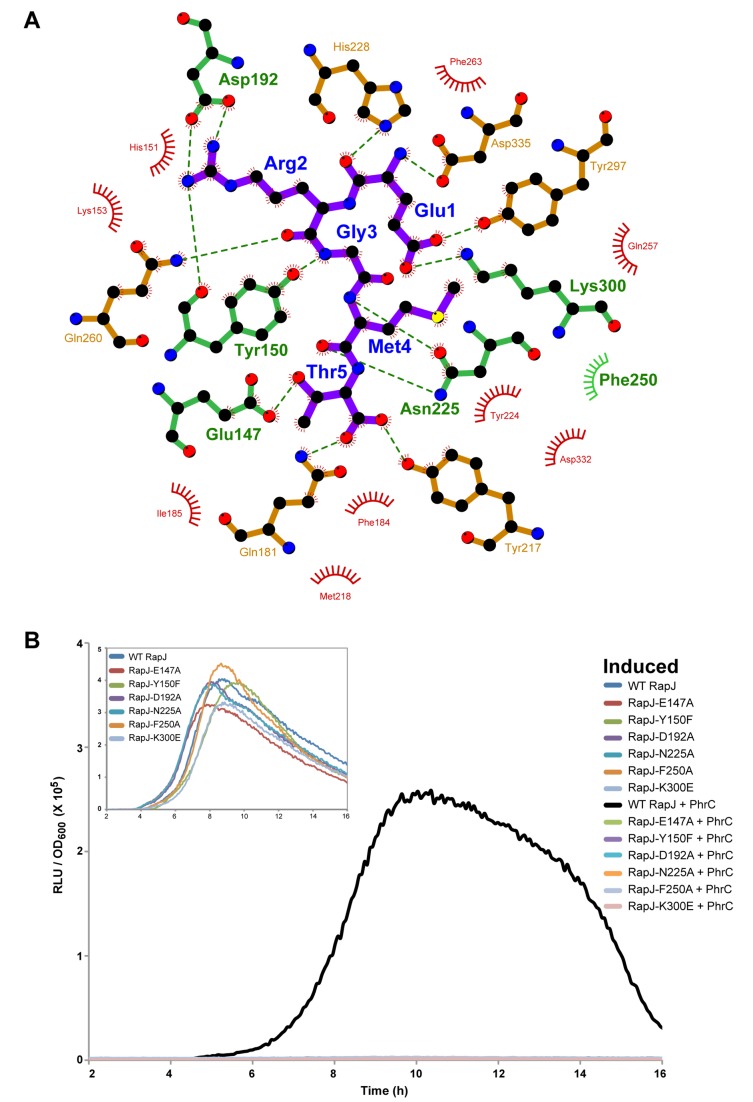
Functional analysis of the RapJ-PhrC interface. (A) Schematic representation of the RapJ-PhrC interface. RapJ residues are depicted with green and brown bonds. Hydrogen bonds are depicted as dashed green lines. Red and green semicircles with radiating lines depict hydrophobic contacts between RapJ and PhrC. Individual substitution mutations were introduced at the positions depicted with green bonds or green semicircles with radiating lines (A), and the mutant proteins were evaluated in the P*spoIIG::luc* reporter assay (B). Each curve is representative of at least three independent experiments performed in duplicate. The inset panel shows that P*spoIIG::luc* strains exhibited similar Spo0A∼P activity in the absence of induced RapJ. PhrC was used at 600 µM. RLUs, Relative Luminescence Units. The schematic was produced with LIGPLOT [60].

To explore the physiological importance of the RapJ-PhrC interactions observed in the RapJ-PhrC structure (Figures 3A and 6A), we systematically mutated RapJ residues that contribute directly to the PhrC binding surface and analyzed the mutants for sensitivity to PhrC using the in vivo P*spoIIG* luciferase reporter assay (Figure 6A and 6B). The vast majority of mutations in the RapJ-PhrC interface resulted in RapJ proteins that were insensitive to PhrC. RapA and RapC mutations that resulted in a loss of sensitivity to PhrA or PhrC, respectively, were previously identified [10],[19],[24],[36]. These mutations are in residues equivalent to RapJ residues D192, Y224, N225, H228, Q260, and V259 (Figure S5). The RapJ-PhrC crystal structure shows that RapJ residues D192, Y224, N225, H228, and Q260 are buried in the PhrC interface (Figure 6A). While V259 is not buried in the PhrC interface, we speculate that a mutation here could affect entry of Phr peptides into the binding pocket. RapJ-PhrC interface mutations E147A, Y150F, D192A, N225A, F250A, and K300E resulted in a complete loss of sensitivity to PhrC (Figure 6A and 6B) and, together with previous mutagenesis studies [10],[19],[24],[36], confirm the biological importance of the crystallographically identified RapJ-PhrC interface.

### Functional Analysis of Intramolecular RapJ Contacts Driven by PhrC Binding

**Figure 7:**
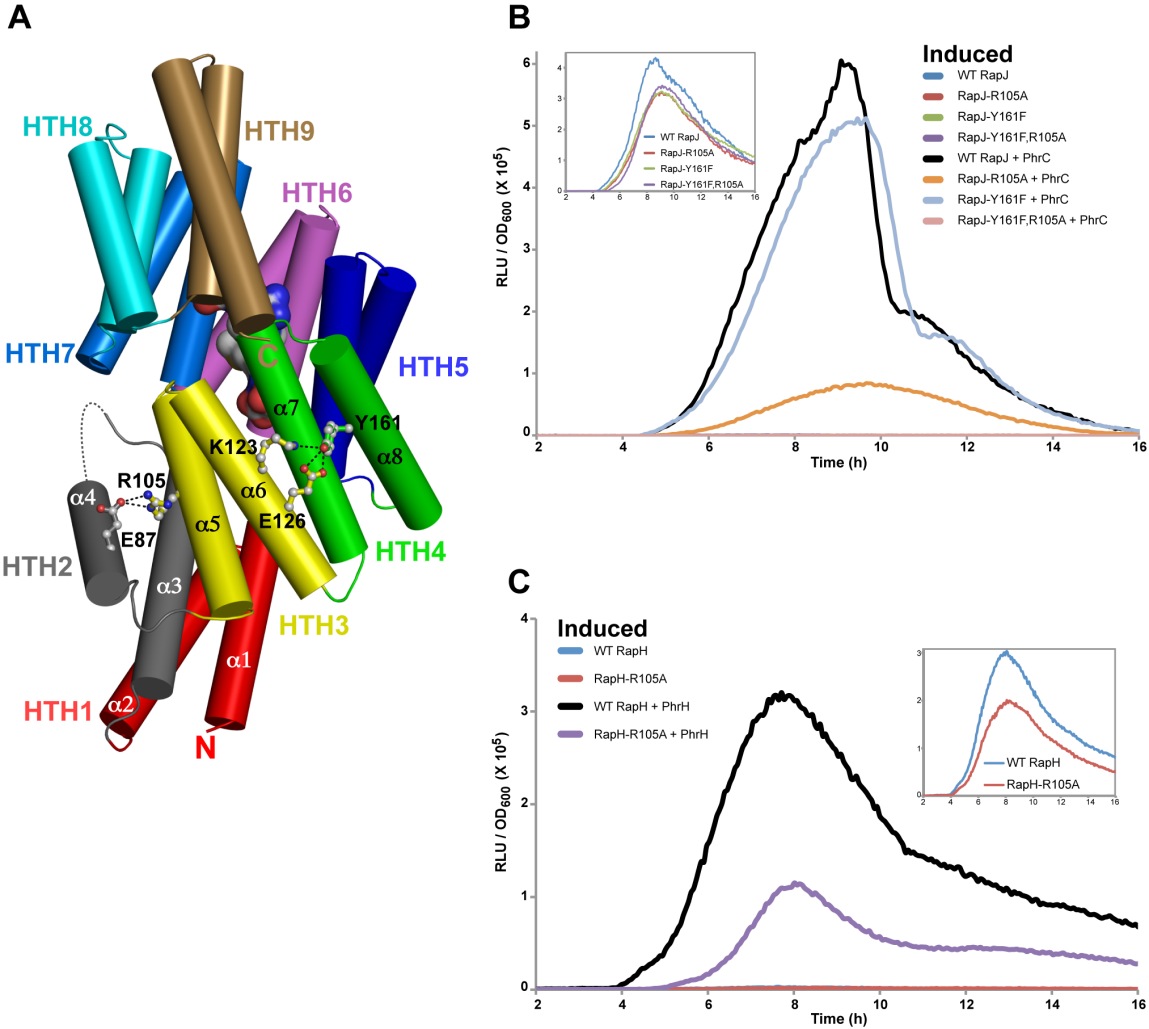
Functional analysis of intramolecular RapJ contacts driven by PhrC binding. (A) The salt bridge formed between RapJ residues Arg105 and Glu87, and the bonds between RapJ residues Glu126, Lys123, and Tyr161, in the RapJ-PhrC structure are depicted. RapJ is depicted in cartoon format, and the surface of PhrC is shown. In vivo activity of the RapJ (B) and RapH mutants (C) containing mutations targeting the Phr-dependent intramolecular Rap protein surfaces. Rap activity was measured as a function of P*spoIIG*::*luc* expression (see Figure S1A and S1B). The inset panels show the Spo0A∼P activity of the P*spoIIG::luc* strains in the absence of induced RapJ or RapH. Each curve is representative of at least three independent experiments performed in duplicate. RLUs, Relative Luminescence Units.

PhrC binding to RapJ creates not only intermolecular RapJ-PhrC contacts but also new intramolecular contacts between regions of RapJ distant from the PhrC binding site. Comparison of the RapJ-PhrC, RapI, RapH-Spo0F, and RapF-ComA_C_ structures enabled us to recognize PhrC-induced intramolecular RapJ contacts. We hypothesized that some of these RapJ contacts, in particular the contacts between HTH folds, are critical for stabilizing the catalytically inactive PhrC-bound conformation. To identify intramolecular RapJ contacts that stabilize the PhrC-bound conformation, we mutated RapJ residues that are distant from the PhrC binding site but form new intramolecular contacts upon PhrC binding and determined whether the mutant proteins were constitutively active in the presence of PhrC in vivo (Figure 7A and 7B). More specifically, we targeted for mutagenesis residues that mediate PhrC-dependent contacts between HTH folds (e.g., a salt bridge formed between HTH2 and HTH3) at a distance from the PhrC binding site.

RapJ residue R105 (located in helix-α5 in HTH3) forms a salt bridge with E87 (located in helix-α4 in HTH2) (Figure 7A). RapJ-R105A displayed wild-type Spo0F phosphatase activity, and RapJ-R105A was largely insensitive to the effects of PhrC (Figure 7B). RapJ residue E87 interacts with the Rap protein 3-helix bundle when the Rap proteins are bound to Spo0F or ComA. The RapJ-E87A mutant displayed a severe phosphatase defect (unpublished data), and RapJ-E87 could not be evaluated for PhrC sensitivity. RapJ residue Y161 (located in helix-α8 in HTH4) interacts with residues K123 and E126 (both located in helix-α6 in HTH3) (Figure 7A). RapJ-Y161F displayed wild-type Spo0F phosphatase activity and wild-type sensitivity to PhrC (Figure 7B). However, the double mutant RapJ-R105A,Y161F displayed wild-type phosphatase activity and complete insensitivity to PhrC (Figure 7B). Consistent with the fact that these contacts are distant from the PhrC binding site, using MALDI-TOF and MALDI-TOF/TOF tandem mass spectrometry we detected PhrC complexed with RapJ-R105A,Y161F following SEC of RapJ-R105A,Y161F incubated with PhrC (Figure S6). RapJ residues R105 and Y161 are highly conserved among the *B. subtilis* Rap proteins (Figure S5), and the fact that RapJ-R105A,Y161F is competent to bind PhrC but insensitive to its inhibitory effects confirms the biological importance of the RapJ conformation observed in the RapJ-PhrC crystal structure. Furthermore, consistent with the possibility that the RapJ conformation observed in the RapJ-PhrC crystal structure is the inactive conformation adopted by other Rap proteins upon binding to Phr peptides, RapH-R105A was insensitive to the effects of PhrH in vivo (Figures 7C and S1B).

## Discussion

Sequence and structural analysis shows that the Rap proteins are repeat proteins consisting of nine HTH TPR or TPR-like folds, which pack together to form a right-handed superhelical TPR domain (Figure 3A and 3B) [8],[22],[28]. TPR proteins are the most common repeat proteins in bacteria, comprising 14% of all bacterial repeat proteins, which make up greater than 5% of the bacterial proteome [37]. Repeat proteins like the Rap proteins form extended structures; thus, they have a larger surface area to volume ratio than globular proteins. Due at least in part to this large surface area to volume ratio, repeat proteins commonly mediate protein–protein and protein–peptide interactions.

It is accepted dogma that repeat proteins do not undergo large conformational changes upon protein or peptide ligand binding (for review, see [38]). This concept has been widely accepted simply because there were no data suggesting that repeat proteins are particularly flexible or undergo large conformational changes. Upending this widely held belief, comparison of the structures of RapI alone, RapJ-PhrC, RapH-Spo0F, and RapF-ComA_C_ reveals that Rap proteins can undergo enormous conformational changes (Figure 5 and Movie S1). In fact, as discussed below, our data show that Rap proteins can exist in single-domain or dual-domain forms.

When complexed with a target such as Spo0F or ComA, Rap proteins have a distinct N-terminal 3-helix bundle, helix-containing linker region, and C-terminal TPR domain (Figure 5). In the case of RapF, the 3-helix bundle and linker region form the ComA_C_ binding surface. In the case of RapH, the C-terminal TPR domain and 3-helix bundle make critical contacts with Spo0F; in fact, the 3-helix bundle contains the catalytic glutamine that inserts into the Spo0F active site. Comparison of the RapJ-PhrC, RapH-Spo0F, and RapF-ComA_C_ structures reveals the mechanism of Phr regulation. The Phr-induced conformational change (detailed below) not only simultaneously results in a total rearrangement of both the ComA_C_ and Spo0F binding sites, but also (1) buries (renders inaccessible) RapJ residues corresponding to RapF residues that bind ComA_C_, including Phe24 and Leu67, which were previously shown to mediate critical interactions with ComA_C_ [28], and (2) splits and displaces the Spo0F binding surface on the 3-helix bundle (including the catalytic glutamine) and TPR domain to opposite sides of the Rap protein where they cannot interact concurrently with Spo0F.

In both the RapH-Spo0F and RapF-ComA_C_ structures, the Rap linker region helix lies at a ∼45° angle to the 3-helix bundle (Figure 5). In comparison to the Spo0F and ComA_C_ bound structures, in both the RapI and RapJ-PhrC structures, the N-terminal 3-helix bundle has rotated ∼180° and the linker region helix has rotated ∼135° (Figure 5). Together, the rotation of the 3-helix bundle and linker region helix creates two HTH folds (HTH1 and HTH2; Figure 5) that pack against the existing C-terminal TPR domain, resulting in the extension of the TPR domain fold by two HTH repeats. To our knowledge, this represents the first example of conformational change-induced repeat domain expansion.

Structural alignment of the RapI and RapJ-PhrC structures shows that RapJ in the PhrC bound conformation is compressed along the TPR superhelical axis, causing the disappearance of the concave groove that exists in the structures of RapI alone, RapH-Spo0F, and RapF-ComA_C_. New intramolecular RapJ contacts that form as a result of the PhrC-induced compression stabilize the RapJ closed conformation and are necessary for PhrC inhibition of RapJ Spo0F phosphatase activity. The fact that the R105A mutation resulted in a severe loss of RapJ and RapH sensitivity to PhrC and PhrH, respectively, suggests that this residue is a hotspot contributing significantly to the binding energy of the intramolecular interface formed in the closed, Phr peptide-bound conformation.

We speculate that the N-terminal 3-helix bundle moves into the intermediate open conformation when Phr peptides dissociate from Rap proteins in the closed conformation or when Spo0F or ComA dissociate from Rap proteins in the fully open conformation (Figure 5). While additional studies are required to determine whether the conformation of the 3-helix bundle and linker region in the RapI alone crystal structure represents a stable Rap protein conformation adopted in the absence of Phr peptide or target protein, existing data suggest that this is not likely to be the case. More likely is the possibility that the structure of RapI alone depicts one of many transient conformations that the 3-helix bundle and linker region can adopt in the absence of a Phr peptide or target protein. In fact, as mentioned above, there are two copies of RapI in the crystallographic asymmetric unit, and while the model of one molecule is relatively complete and includes the majority of the 3-helix bundle and linker region, there was in fact insufficient electron density to build the N-terminal 3-helix bundle and linker region in the other molecule (see Materials and Methods). In the more complete RapI model, where the N-terminal 3-helix bundle and linker region are included, the N-terminal 3-helix bundle, linker region, and C-terminal TPR domain together bury 857 Å^2^ surface area at their interface. However, consistent with the idea that the structure of RapI depicts one of many transient conformations, we note that the position of the N-terminal 3-helix bundle and linker region in the more complete RapI model is likely influenced by crystal contacts with symmetry-related copies of RapI. The above results suggest that in the absence of a bound target such as Spo0F or ComA, or a bound Phr peptide such as PhrC, the N-terminal 3-helix bundle and linker region may adopt different conformations relative to the C-terminal TPR domain.

What is the structural basis of Rap-Phr binding and selectivity? The residue at position −4 from the C-terminus of the Phr penta and hexapeptides is basic (Arg or Lys) in all of the identified *B. subtilis* Phr peptides; it is Arg in eight of nine *B. subtilis* Phr peptides, and it is Lys in only one instance, PhrG [9]. The RapJ-PhrC structure revealed that RapJ residue Asp192 forms a salt-bridge with PhrC residue Arg2 (Figure 6A). With the exception of RapG, every *B. subtilis* Rap protein contains Asp at the position structurally equivalent to RapJ Asp192; in RapG, this residue is Glu (Figure S5). We propose that the salt bridge between RapJ-D129 and PhrC-R2 is conserved in every *B. subtilis* Rap-Phr complex. This hypothesis is supported by previous analysis of Rap proteins containing mutations in the position equivalent to RapJ Asp192 [10],[19],[24],[36], as well as by studies analyzing the effects of substitution mutations in Phr peptides at the position equivalent to PhrC Arg2 [7]. We propose that Phr peptides recognize their cognate Rap protein by first scanning the surface for a pocket of complementary shape. Second, the Rap-Phr salt bridge equivalent to the interaction of RapJ Asp192 and PhrC Arg2 is a hotspot contact that anchors the peptide-receptor complex.

With the exception of the basic residue conserved at the position −4 from the C-terminus, the remaining *B. subtilis* Phr residues are poorly conserved and each could contribute to the determination of Rap-Phr interaction specificity. While there are not yet enough known Rap-Phr pairs to use multiple sequence alignment to identify covarying residues at the Rap-Phr interface, we have identified a number of *B. subtilis* Rap-Phr interactions that likely contribute to the determination of interaction specificity. For example, the PhrC residue Glu1 sidechain hydrogen bonds with the Rap Tyr297 and Lys300 sidechains (Figure 6). Sequence analysis of the *B. subtilis* Rap-Phr pairs shows that when the Phr residue equivalent to PhrC residue Glu1 is Glu or Asp, then the position equivalent to RapJ Tyr297 is Tyr and the position equivalent to RapJ Lys300 is Lys. However, when the Phr residue equivalent to PhrC residue Glu1 is Ala or Ser, then the Rap position equivalent to RapJ Tyr297 is Phe and the position equivalent to RapJ Lys300 is Leu. Similarly, the PhrC Gly3 mainchain nitrogen hydrogen bonds with the RapJ Tyr150 sidechain (Figure 6), and this appears to be the case for every *B. subtilis* Rap-Phr pair with the following exception. In the RapK-PhrK pair the Phr residue equivalent to PhrC residue Gly3 is Pro, and RapK encodes Thr at the position equivalent to RapJ Tyr150. Likewise, the PhrC Met4 mainchain nitrogen and carbonyl form hydrogen bonds with the RapJ Asn225 sidechain (Figure 6), and this Asn is conserved in every *B. subtilis* Rap protein. Thus, this Rap-Phr interaction contributes to the binding energy but not to the determination of binding specificity in *B. subtilis*. Finally, we note that when the Phr residue equivalent to PhrC residue Met4 is Met, then the positions in RapB, RapC, and RapF equivalent to RapJ Tyr224 and Phe250 are Tyr and Phe, respectively (Figure 6). Ongoing computational modeling and peptide docking studies guided by the RapJ-PhrC structure as well as complementary genetic and biochemical analysis of the calculated Rap-Phr interactions will test the importance of the above observations and reveal more broadly the Rap and Phr contacts that dictate Rap-Phr interaction specificity at the atomic level.

Finally, analogous to antibodies and their hypervariable complementarity determining regions, repeat proteins such as the TPR proteins can be imagined as a structurally conserved backbone decorated with functional residues. This is exemplified by the variable lymphocyte receptors (VLRs) in jawless fish. Instead of the immunoglobulin-based antigen receptors created by V(D)J recombination in jawed vertebrates, the VLRs result from the combinatorial assembly of leucine-rich repeats [39]. Studies of VLRs and numerous other repeat proteins such as TPR and ankyrin repeat proteins have provided tremendous insight into repeat protein function while also advancing our ability to evolve or engineer repeat proteins displaying new functions [40]–[48]. In fact, repeat proteins are now widely used as scaffolds for the development of designed affinity reagents—for example, Designed Ankyrin Repeat Proteins (DARPins) and TPR-based recognition modules (T-Mods)—which can substitute for antibodies in chromatographic, diagnostic, co-crystallization, and therapeutic applications. In comparison to antibodies, repeat proteins can offer a number of advantages including elevated solubility, high production yields in microbial expression systems, protease resistance, and thermal stability. Antibodies are particularly difficult to manufacture since they are glycosylated and contain disulphide bonds. The stability of even truncated forms of antibodies, such as scFv and Fab fragments, relies on the formation of intradomain disulphide bonds [49], also limiting their application. Due to the development of powerful selection techniques (e.g., ribosome display), the effort required to generate alternative binding reagents with prescribed target-binding specificity is quickly approaching that required to create conventional antibodies [40].

Both the oligopeptide binding site on the Rap protein TPR concave surface and the multiple target protein binding sites on the Rap protein TPR convex surface could be engineered to bind different oligopeptides and proteins, respectively. Furthermore, the RapJ-PhrC X-ray crystal structure shows a channel leading into the oligopeptide binding site, suggesting that the RapJ peptide interaction surface could be engineered to bind the flexible C-terminus of a target protein. As discussed above, prior to our studies, repeat proteins were not known to undergo large ligand-induced conformational changes. Consequently, the use of peptides or other ligands to regulate the target binding of designed affinity reagents has not been previously explored. We propose that Rap proteins could serve as scaffolds for engineering or evolving ligand-switchable TPR-based affinity reagents.

## Materials and Methods

### Protein Production for X-Ray Crystallography

*RapJ* was amplified from *B. subtilis* strain 168 genomic DNA using Phusion High-Fidelity DNA Polymerase and the primer pair RapJ-Fwd and RapJ-Rev (Table S3). The PCR product was cloned into the SapI and XhoI sites of pTB146 using the In-Fusion method (Clontech) to give pTB146J [50]. His-Sumo-RapJ was overexpressed in *E. coli* strain BL21(DE3) by first growing the cells at 37°C in LB medium containing 100 µg/ml ampicillin to OD_600_ = 0.6 and then inducing expression with 0.1 mM isopropyl β-D thiogalactopyranoside (IPTG) for 16 h at 16°C. All subsequent purification steps were carried out at 4°C. The cells were collected by centrifugation and lysed in buffer A (20 mM Tris-HCl, pH 8.0, 250 mM NaCl, 50 mM KCl, 10 mM MgCl_2_, 10 mM β-ME, 10% glycerol) supplemented with 1 µM Pepstatin, 1 µM Leupeptin, 20 µg/ml DNase, and 1 mM PMSF. Lysate supernatant was applied to His-60 Ni resin (Clontech) equilibrated in buffer A. The His-60 resin was then washed in buffer A and resuspended in 65 mM Tris-HCl (pH 8.0), 325 mM NaCl, 35 mM KCl, 7 mM MgCl_2_, 3.5 mM DTT, 10% glycerol, and 0.2% NP-40. SUMO protease was then added at 4 mg/ml His-60 resin and incubated at 4°C for 16 h. RapJ contained no heterologous residues following removal of the N-terminal His-Sumo fusion. RapJ was eluted with buffer A and diluted 3-fold with buffer B (20 mM Tris-HCl, pH 8.0, 10 mM MgCl_2_, 5 mM DTT, and 10% glycerol), passed through a 0.45 µm filter, and loaded onto an anion exchange column (Source 15Q; GE Healthcare) equilibrated in buffer B containing 50 mM KCl. RapJ was then eluted in a 50–1,000 mM KCl gradient of buffer B. Fractions containing RapJ were pooled, concentrated by ultrafiltration through a 30 kDa filter, and further purified by gel filtration using a Superdex 200 (GE Healthcare) 16/70 column equilibrated in buffer C (20 mM Tris-HCl, pH 8.0, 150 mM KCl, 5 mM MgCl_2_, and 5 mM DTT). RapJ was concentrated to 1.58 mM and stored at −80°C. Expression of selenomethionyl RapJ was in *E. coli* strain B834(DE3) grown in M9 medium [51]. A total of 10 mM dithiothreitol was present throughout the purification, which was otherwise performed as described above for native RapJ. Selenomethionyl RapJ was concentrated to 1.35 mM before storing at −80°C.

*RapI* was amplified from *B. subtilis* str. 168 genomic DNA using primers RapI_Fwd_Infusion and RapI_Rev_XhoI and cloned into the SapI and XhoI sites of pTB146 using the In-Fusion method to give pTB146I (Table S3). *ImmA* was amplified from *B. subtilis* str. 168 genomic DNA using primers ImmA-pBB-NdeI-Fwd and ImmA-pBB-EcoRI_rev and cloned into NdeI and EcoRI sites of pBB75 by the In-Fusion cloning to give pBBA. *ImmR* was amplified from *B. subtilis* str. 168 genomic DNA using primers ImmR1_F_pCOLANcoI_Inf and ImmR1_R_pCOLANotI_Inf and cloned into NcoI and NotI sites of pACYCDuet-1 (Novagen) by the In-Fusion method to give pACYCR.

His-Sumo-RapI, untagged ImmA, and untagged ImmR were overexpressed following co-transformation of pTB146I, pBBA, and pACYCR in *E. coli* strain BL21(DE3) by first growing the cells at 37°C in LB medium containing 100 µg/ml ampicillin, 30 µg/ml kanamycin, and 17 µg/ml chloramphenicol to OD_600_ = 0.8 and then inducing expression with 1 mM IPTG for 16 h at 18°C. Cells were lysed in Buffer D (20 mM Tris, pH 8.0, 250 mM NaCl, 50 mM KCl, 20 mM βME, 10 mM MgCl_2_, and 10% glycerol) supplemented with 1 µM Pepstatin, 1 µM Leupeptin, 20 µg/ml DNase, and 1 mM PMSF. Lysate supernatant was applied to His-60 resin equilibrated in buffer D. His-Sumo-RapI bound to the His-60 resin, while ImmA and ImmR were not retained. The Ni resin was then washed in buffer D and eluted with buffer D containing 20, 50, 100, 200, or 500 mM imidazole. The 50–500 mM imidazole-containing fractions were pooled and 50 µg SUMO protease was then added per 1 mg of total protein in 40 mM Tris, pH 8.0, 0.2% NP40, 50 mM NaCl, 190 mM KCl, 1 mM DTT, 16 mM βME, 8 mM MgCl_2_, and 8% glycerol and incubated at 4°C for 16 h. RapI contained no heterologous residues following removal of the N-terminal His-Sumo fusion. Protein was centrifuged at 15,000 rpm for 10 min to remove precipitated protein, passed through a 0.45 µm filter, diluted 3-fold with buffer E (20 mM Tris-HCl, pH 8.0, 10 mM MgCl_2_, 5 mM DTT, and 10% glycerol) and loaded onto a Source15Q column equilibrated in buffer E containing 50 mM KCl. RapI was then eluted in a 50–1,000 mM KCl gradient of buffer E. Fractions containing RapI were pooled, concentrated by ultrafiltration through a 30 kDa filter, and further purified by gel filtration using a Superdex 200 16/70 column equilibrated in buffer F (20 mM Tris-HCl, pH 8.0, 150 mM KCl, 5 mM MgCl_2_, and 5 mM DTT). RapI was concentrated to 608 µM and stored at 4°C for less than 10 d prior to its use in crystallization experiments.

### Crystallization and Diffraction Data Collection

Native RapJ-PhrC crystals were produced by the vapor diffusion method at 20°C using a 1∶1 mixture of RapJ-PhrC (250 µM RapJ and 1.24 mM PhrC in 17.7 mM Tris-HCl, pH 8.0, 133.2 mM KCl, 4.4 mM DTT, 4.4 mM MgCl_2_, and 2% Benzamidine hydrochloride hydrate) and well solution (8.8% [w/v] PEG 3000, 290 mM magnesium chloride, and 100 mM sodium cacodylate, pH 6.4). RapJ-PhrC crystals were soaked and cryoprotected in mother liquor solution containing 3%, 7.5%, and 14% glycerol for ∼5 s each followed by 4 h soaking in mother liquor solutions containing 20% glycerol.

RapJ-PhrC crystals containing the selenomethionyl derivatized RapJ protein were produced by the vapor diffusion method at 20°C using a 1∶1 mixture of RapJ-PhrC (250 µM RapJ and 1.24 mM PhrC in 17.7 mM Tris-HCl, pH 8.0, 133.2 mM KCl, 8.8 mM DTT, 4.4 mM MgCl_2_, and 2% Benzamidine hydrochloride hydrate) and well solution (8.8% [w/v] PEG 3000, 250 mM magnesium chloride, and 100 mM sodium cacodylate, pH 5.4). RapJ-PhrC crystals were soaked and cryoprotected in mother liquor solution containing 3%, 7.5%, and 14% glycerol for ∼5 s each followed by 5 min soaking in mother liquor solutions containing 20% glycerol. Single-wavelength anomolous dispersion (SAD) and native data on nitrogen-cooled crystals were collected at NSLS beamline X29 and processed using the HKL software package [52].

RapI crystals were produced by the vapor diffusion method at 20°C using a 1∶1 mixture of RapI (145 µM in 20 mM Tris-HCl, pH 8.0, 150 mM KCl, 5 mM DTT, 5 mM MgCl_2_) and well solution (17% [w/v] PEG 3350, 200 mM lithium nitrate, and 100 mM Tris-HCl, pH 7.8). RapI crystals were soaked and cryoprotected in mother liquor solutions containing 3.0%, 7.5%, and 14.0% glycerol for ∼5 s each followed by 5 min soaking in mother liquor solutions containing 20% glycerol. Native data on nitrogen-cooled crystals were collected at NSLS beamline X29A and processed using the HKL software package.

### Structure Determination and Refinement

The RapJ-PhrC crystal structure was determined by the SAD method using crystals of selenomethionyl RapJ bound to PhrC that were isomorphous to the native RapJ-PhrC crystals. PHENIX (AutoSol) was used to locate heavy atom positions, calculate phases, and generate an initial model at 2.21 Å resolution [53]. This model was then refined against 2.16 Å native data in PHENIX. The final model was generated through iterative cycles of building in COOT [54] and refinement in PHENIX. The RapJ and PhrC models were built de novo into the SAD-phased map. The earliest rounds of refinement in PHENIX employed simulated annealing as well as individual atomic coordinate and individual B-factor refinement. The later rounds of refinement in PHENIX employed individual atomic coordinate and individual B-factor refinement, as well as a TLS model whose initial parameters were guided by the TLS Motion Determination (TLSMD) server [55]. During the final rounds of refinement in PHENIX, the stereochemistry and ADP weights were optimized—that is, the weights yielding the lowest R_free_ value were used for refinement. PhrC molecules were added only after the RapJ models were built, and then water molecules were added. Insufficient electron density was observed for the following residues and they were omitted from the model: RapJ_A_ 1–5, 72–77, and 90–92; RapJ_B_ 1–6 and 72–77. Two chlorine atoms were built into clear electron density during the final stages of refinement. The RapI crystal structure was determined by molecular replacement. A conserved region of RapJ consisting of residues 217–365 was used as an initial search model for molecular replacement. Phenix.mr_rosetta and RapI sequence alignment was then used to rebuild the starting model, resulting in 1,000 new models. The Phaser LLG score was used to identify the best model. The model identified here was then extended to include residues 175365, and the model was then subjected to another cycle of rebuilding using phenix.mr_rosetta. Phaser was then used to identify the best model and also place a second copy in the crystallographic asymmetric unit. Arp/wArp was then used to improve the model and map. Insufficient electron density was observed for the following residues, and they were omitted from the model: RapI_A_ 1–100 and 375–391; RapI_B_ 1–13, 73–77, and 378–391. Ramachandran statistics were calculated in Molprobity [56]. Molecular graphics were produced with PyMOL [57].

### Construction of *B. subtilis* P*spoIIG*::*luc* Reporter Strains

The *B. subtilis* IS75 *rapJ* markerless deletion strain was constructed by amplifying a region upstream of *rapJ* using the primer pair ΔrapJ_5′_Inf_F and ΔrapJ_5′_Inf_R and a region downstream of *rapJ* using primer pair ΔrapJ_3′_Inf_F and ΔrapJ_3′_Inf_R (Table S3). To generate pMiniRapJ, In-Fusion cloning was used to simultaneously ligate the PCR products and insert them into the EcoRI and SalI sites of pMini-MADII (a kind gift from Dan Kearns, Indiana University), which carries a temperature-sensitive origin of replication and an erythromycin resistance cassette. The Δ*rapJ*, P*spoIIG*::*luc* strain VP068 was constructed by transforming pMiniRapJ into the P*spoIIG*::*luc* strain PP533 (a kind gift from David Dubnau, PHRI). Growth on LB agar containing 0.5 µg/ml erythromycin and 2.5 µg/ml lincomycin at the restrictive temperature (37°C) that inhibits plasmid replication selected single-crossover events integrating pMiniRapJ into the chromosome. To evict the plasmid, the strain was incubated in 3 ml LB broth at a permissive temperature for plasmid replication (22°C) for 14 h, diluted 30-fold in fresh LB broth, and incubated at 22°C for another 8 h. Dilution and outgrowth were repeated two more times. Cells were then serially diluted and plated on LB agar at 37°C. Fifty individual colonies were patched in duplicate on LB agar alone and LB agar containing 0.5 µg/ml erythromycin and 2.5 µg/ml lincomycin to identify colonies that had evicted the plasmid. Chromosomal DNA from colonies that had excised the plasmid was purified and screened by PCR using primers ΔrapJ_5′_Inf_F and ΔrapJ_3′_Inf_R to determine which isolate had retained the Δ*rapJ* allele. The *rapJ* deletion was confirmed by Western blotting using anti-RapJ rabbit antisera.

To generate VP068 strains expressing wild-type and mutant RapJ proteins, *rapJ* was PCR amplified from *B. subtilis* strain IS75 chromosomal DNA using primers pHyspank_rapJ_F and pHyspank_rapJ_R. The PCR product was cloned into the SalI and SphI sites in pDR111 (a kind gift from D. Rudner, Harvard Medical School) using the In-Fusion method. The resulting plasmid, pDRJ1, was then mutagenized using the appropriate mutagenic primers (Table S3) and either the ChangeIT Mutagenesis (USB) or Quikchange II XL Mutagenesis (Agilent technologies) protocols. RapH-R105A was similarly generated using pDRH1 [22]. DNA sequencing confirmed that the pDRH1- and pDRJ1-derived plasmids were free of mutations other than those introduced by site-directed mutagenesis. The pDRJ1- and pDRH1-derived plasmids were then transformed into the Δ*rapJ*, P*spoIIG*::*luc* strain VP068 or the Δ*rapHphrH*, P*spoIIG*::*luc* strain BD5035 [22], respectively. Double-crossover recombination at the VP068 *amyE* locus yielded strains that express wild-type or mutant RapJ proteins under the control of the IPTG-inducible hyperspank promoter. Double-crossover recombination at the BD5035 *amyE* locus yielded strains that express wild-type or mutant RapH proteins under the control of the IPTG-inducible hyperspank promoter.

### Luciferase Bioassays

The reporter strains were grown in LB medium to OD_600_≈2, centrifuged, and resuspended in fresh Sporulation Medium (DSM) [58] to OD_600_ = 2. The cultures were then diluted 20-fold in fresh DSM. For the RapJ assays, the cultures were supplemented with 60 µM IPTG and PhrC at the concentrations indicated. For the RapH assays, the cultures were supplemented with 100 µM IPTG and 20 µM PhrH. 200 µl were dispensed per well in duplicate in 96-well black plates (Corning). 10 µl of luciferin was added to each well at a final concentration of 4.7 mM. The cultures were then incubated at 37°C under agitation in a PerkinElmer Envision 2104 Multilabel Reader. The plate lids were heated to 38°C to avoid condensation. Relative Luminescence Unit (RLU) and OD_600_ were measured at 3 min intervals.

### Protein Production for Phosphatase Assays

RapJ and RapI were overexpressed and purified as described above with the exception that dialysis rather than gel filtration was used to exchange the proteins into 20 mM Tris-HCl (pH 8.0), 150 mM KCl, 5 mM MgCl_2_, 5 mM DTT, and 10% glycerol. RapJ and RapI were stored at −80°C. Spo0F containing a C-terminal fusion to hexahistidine was purified as previously described [59]. KinA containing an N-terminal fusion to hexahistidine was purified as previously described [22].

### Spo0F Labeling and In Vitro Phosphatase Assay

Spo0F labeling and in vitro phosphatase assays were performed as described previously [22] except that final reaction conditions were 32.5 µM DRVGA, 32.5 µM ADRVGA, 310 µM TDRNTT, 310 µM ARNQT, or 310 µM ERGMT; 6.5 µM RapJ or 6.5 µM RapI; 6.0 µM radiolabeled Spo0F∼P and 24 µM Spo0F; and 2.85 µM KinA, 14.55 mM Tris (pH 8.0), 50 mM EPPS (pH 8.5), 0.1 mM EDTA, 100 mM KCl, 23 mM MgCl_2_, 3 mM DTT, 11.6% glycerol, 0.04 µM [γ-^32^P] ATP, and 1 mM ATP.

### Sedimentation Equilibrium Analytical Ultracentrifugation Analysis

SE AUC measurements were carried out as previously described [22] with the following modifications: gel purified RapJ was used at 50 µM, PhrC was used at 500 µM, the samples were prepared in buffer C, and the rotor speed was 13,000 rpm.

### Sample Preparation for MALDI-TOF and MALDI-TOF/TOF

Wild-type RapJ and RapJ-R105A,Y161F were overexpressed and purified as they were for X-Ray Crystallography with the following changes to the protein purification protocol. Subsequent to the SourceQ purification step, the samples were dialyzed against buffer H (20 mM Tris-HCl, pH 8.0, 50 mM KCl, 5 mM MgCl_2_, 5 mM DTT). PhrC was then added to obtain a final concentration of 8.5 mM PhrC and 425 µM wild-type RapJ or RapJ-R105A,Y161F. The RapJ-PhrC complexes were then loaded to a Superdex 200 16/70 column equilibrated in buffer H. The peak fractions were concentrated to 900 µM and stored at −80°C. In the case of the PhrC alone control, PhrC was loaded to a Superdex 200 16/70 column equilibrated in buffer H, and the fractions corresponding to elution volume of the RapJ-PhrC complexes were pooled and analyzed for the presence of PhrC.

### MALDI-TOF and MALDI-TOF/TOF

MALDI-TOF and MALDI-TOF/TOF MS analyses of the above samples were carried out by first diluting them 100-fold with 5% acetonitrile, and then mixing the diluted samples with an equal volume of matrix solution containing 7 mg/ml alpha-cyano-4-hydroxycinnamic acid, 5 mM of ammonium monobasic phosphate, and 0.1% trifluoroacetic acid in 50% acetonitrile. The mixture was spotted onto a MALDI plate and analyzed with a 4800 Proteomics Analyzer tandem mass spectrometer (AB SCIEX, Framingham, MA, USA) in positive ion mode (*m/z* 500–700). Spectra were analyzed using Data Explorer v4.5 (Applied Biosystems). MALDI-TOF/TOF analysis was performed on *m/z* 593.27 (PhrC[M+H]^+^).

### Oligopeptide Synthesis

Synthetic oligopeptides PhrC (NH_2_-ERGMT-COOH), PhrA (NH_2_-ARNQT-COOH), PhrH (NH_2_-TDRNTT-COOH), PhrI-5mer (NH_2_-DRVGA-COOH), and PhrI-6mer (NH_2_-ADRVGA-COOH) were purchased from LifeTein (South Plainfield, NJ) at 95% purity. The lyophilized oligopeptides were reconstituted as 10 mM stocks in H_2_O for use in crystallographic assays and in vitro phosphatase assays, or the oligopeptides were reconstituted as 10 mM stocks in DSM for use in the vivo P*spoIIG*-luciferase reporter assays. Aliquots of the synthetic oligopeptides were stored at −20°C.

### Accession Numbers

Atomic coordinates and structure factors for RapJ-PhrC and RapI have been deposited in the Protein Data Bank under accession codes 4GYO and 4I1A, respectively.

## Supporting Information

Figure S1 P*spoIIG*-luciferase reporter assays. (A) RapJ expression was induced with 60 µM IPTG, and PhrC was used at 600 µM. (B) RapH expression was induced with 100 µM IPTG, and PhrH was used at 20 µM. Each curve is representative of at least three independent experiments performed in duplicate. RLUs, Relative Luminescence Units.
(TIF)

Figure S2 MALDI-TOF and MALDI-TOF/TOF tandem mass spectrometry of SEC-purified RapJ incubated with PhrC.
(TIF)

Figure S3 MALDI-TOF and MALDI-TOF/TOF of PhrC SEC fractions corresponding to the elution volume of the RapJ-PhrC complex analyzed in Figure S2.
(TIF)

Figure S4 SE AUC analysis of RapJ alone (A) and RapJ-PhrC (B). Natural logarithm of absorbance at 285 nm plotted as a function of the radius (distance to the center of the rotor) squared. As described in the text, the slope of the lines fit to the data show that RapJ and RapJ-PhrC are monomeric.
(TIF)

Figure S5 Rap protein sequence alignment. The amino acid sequences of Bacillus Rap proteins previously demonstrated to be regulated by Phr peptides were aligned using the MultipleAlignerClustalW method in STRAP [61]. The residue numbers indicated above the sequences refer to RapJ. The residues in the RapJ-PhrC interface are surrounded by black boxes. Residues marked with black arrowheads were previously shown to render RapA and RapC insensitive to PhrA and PhrC, respectively [10],[19],[24],[36]. Substitutions at the RapJ-PhrC interface residues highlighted by green boxes resulted in a complete loss of sensitivity to PhrC in vivo (Figure 6B). The highly conserved residue RapJ Asp192, which is highlighted by a green box and in bold type, makes a salt bridge with PhrC Arg2. Substitution mutations at residues participating in PhrC-driven RapJ intramolecular contacts (highlighted by red boxes) resulted in a severe loss of sensitivity to PhrC (Figure 7B). Similarly, RapH-R105A was insensitive to PhrH (Figure 7C). The colors of the cylinders representing α-helices correspond to the coloring scheme used in Figures 4, 5, and 7.
(TIF)

Figure S6 MALDI-TOF and MALDI-TOF/TOF tandem mass spectrometry of SEC purified RapJ-R105A,Y161F incubated with PhrC.
(TIF)

Movie S1 A Rap protein morphing between the ligand-free, target-bound, and Phr peptide-bound conformations. Interpolation between the structures of RapI, RapH-Spo0F (PDB ID 3Q15), and RapJ-PhrC was performed using UCSF Chimera [62], and the movie was assembled in PyMOL [57]. PhrC is depicted as magenta sticks, and Spo0F is depicted as a brown cartoon.
(MOV)

Table S1 Data collection and refinement statistics. R_sym_ = Σ_h_ Σ_i_ | I_i_(h)−<I(h)>|/Σ_h_ Σ_i_ I_i_(h), where I_i_(h) is the i^th^ measurement of h and <I(h)> is the mean of all measurements of I(h) for reflection h. R_work_ = Σ ∥F_o_|−|F_c_∥/Σ |F_o_|, calculated with a working set of reflections. R_free_ is R_work_ calculated with only the test set of reflections. Data for the highest resolution shell are given in parentheses. The structures were determined using single crystals. The reflections I(+) and I(−), related by Friedel's Law, were treated as independent for the purpose of the SAD data only.
(DOC)

Table S2 RapI amino acid identity in highly conserved positions lying in the RapH-Spo0F interface [22]. RapA, RapB, RapE, RapH, and RapJ sequences refer to *B. subtilis* Rap proteins. BXA0205 and BA3790 sequences refer to *B. anthracis* Rap proteins. Sequences were aligned in Geneious Pro and analyzed using the ConSurf [63] server as previously described [22].
(DOC)

Table S3 Oligonucleotides.
(DOC)

## Abbreviations

ComA_C_: DNA binding domain of the transcription factor ComA
HTH: helix-turn-helix
Phr: phosphatase regulator
CSF: competence and sporulation factor
Rap: response regulator aspartate phosphatase
REC: receiver
SE AUC: sedimentation equilibrium analytical ultracentrifugation
TPR: tetratricopeptide repeat
