# Supplementary figures and images for: Conformational Change-Induced Repeat Domain Expansion Regulates Rap Phosphatase Quorum-Sensing Signal Receptors
Source: PLoS Biol. 2013 Mar 19;11(3):e1001512. doi: 10.1371/journal.pbio.1001512 (PMC3601965; doi:10.1371/journal.pbio.1001512)

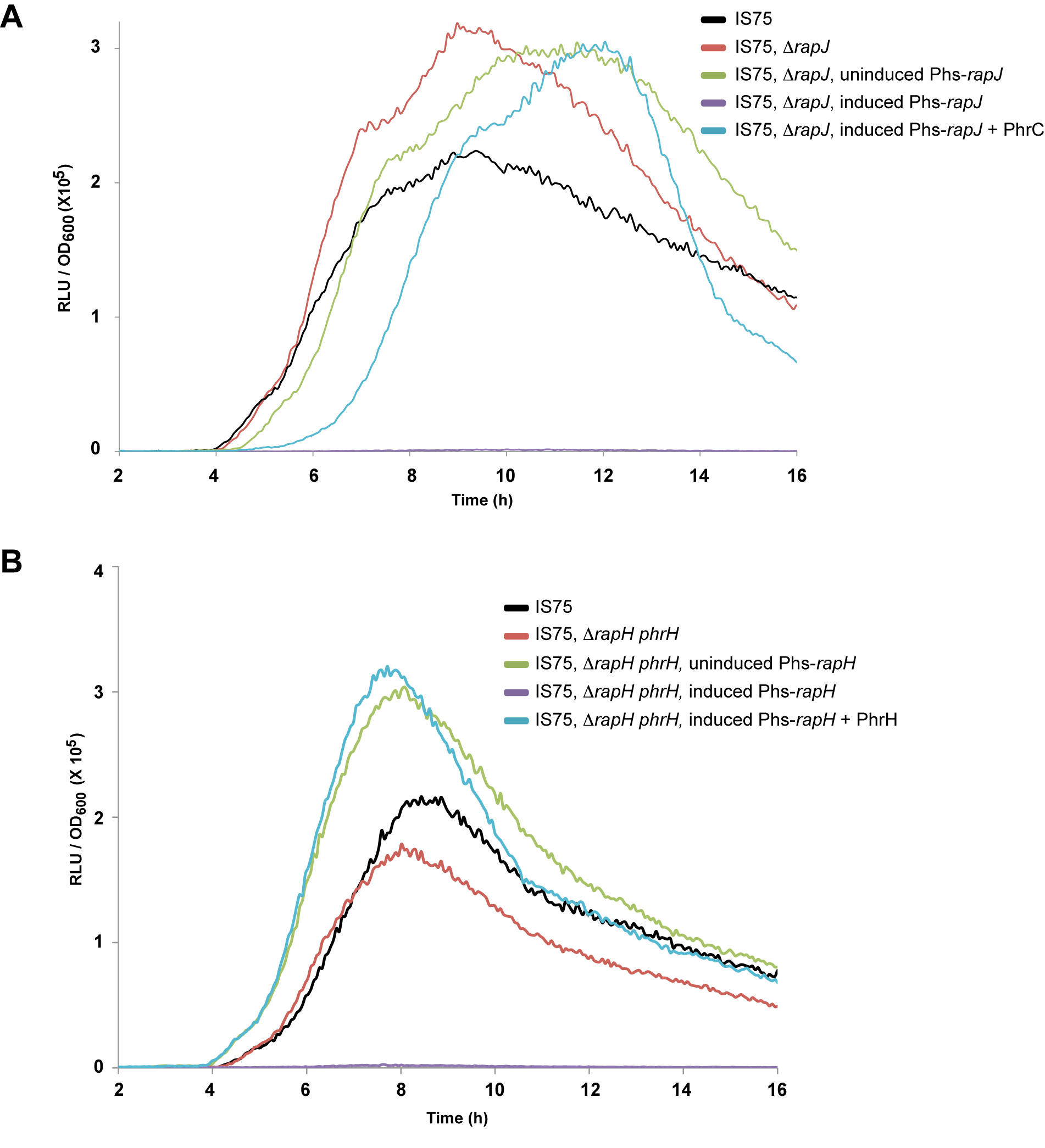

Supplement: Figure S1 — PspoIIG-luciferase reporter assays. (A) RapJ expression was induced with 60 µM IPTG, and PhrC was used at 600 µM. (B) RapH expression was induced with 100 µM IPTG, and PhrH was used at 20 µM. Each curve is representative of at least three independent experiments performed in duplicate. RLUs, Relative Luminescence Units. (TIF) [file pbio.1001512.s001.tif]

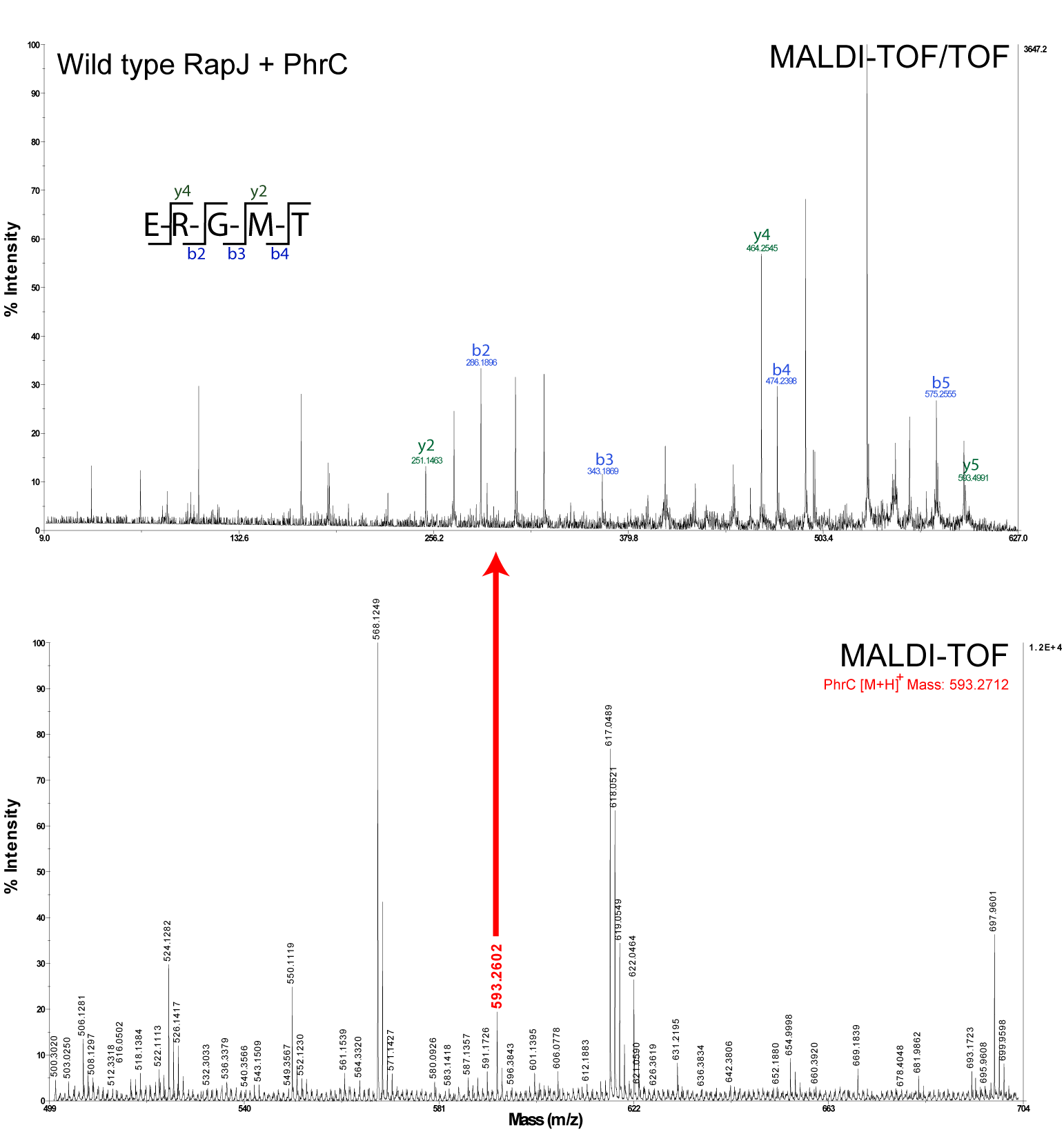

Supplement: Figure S2 — MALDI-TOF and MALDI-TOF/TOF tandem mass spectrometry of SEC-purified RapJ incubated with PhrC. (TIF) [file pbio.1001512.s002.tif]

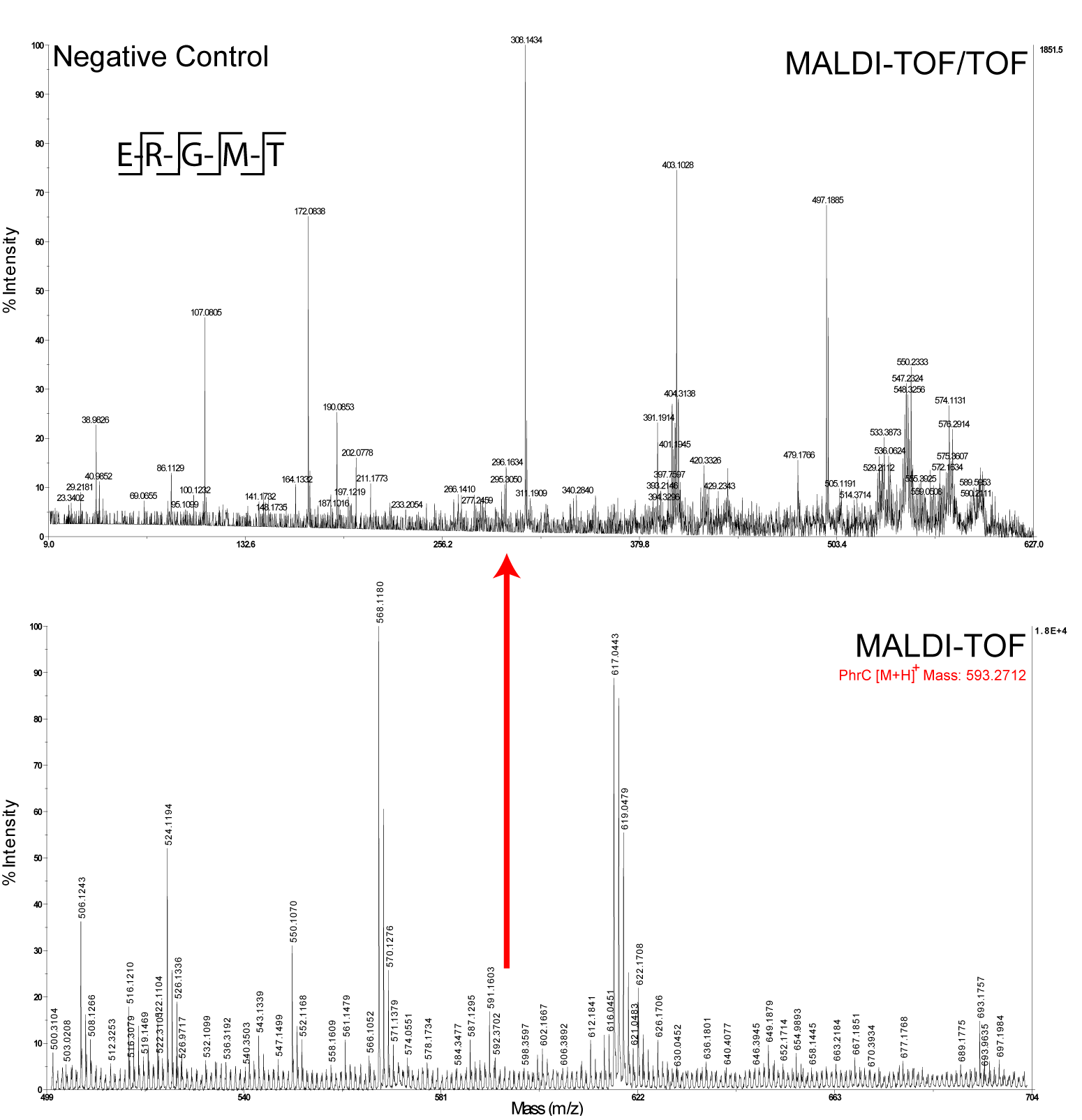

Supplement: Figure S3 — MALDI-TOF and MALDI-TOF/TOF of PhrC SEC fractions corresponding to the elution volume of the RapJ-PhrC complex analyzed in Figure S2. (TIF) [file pbio.1001512.s003.tif]

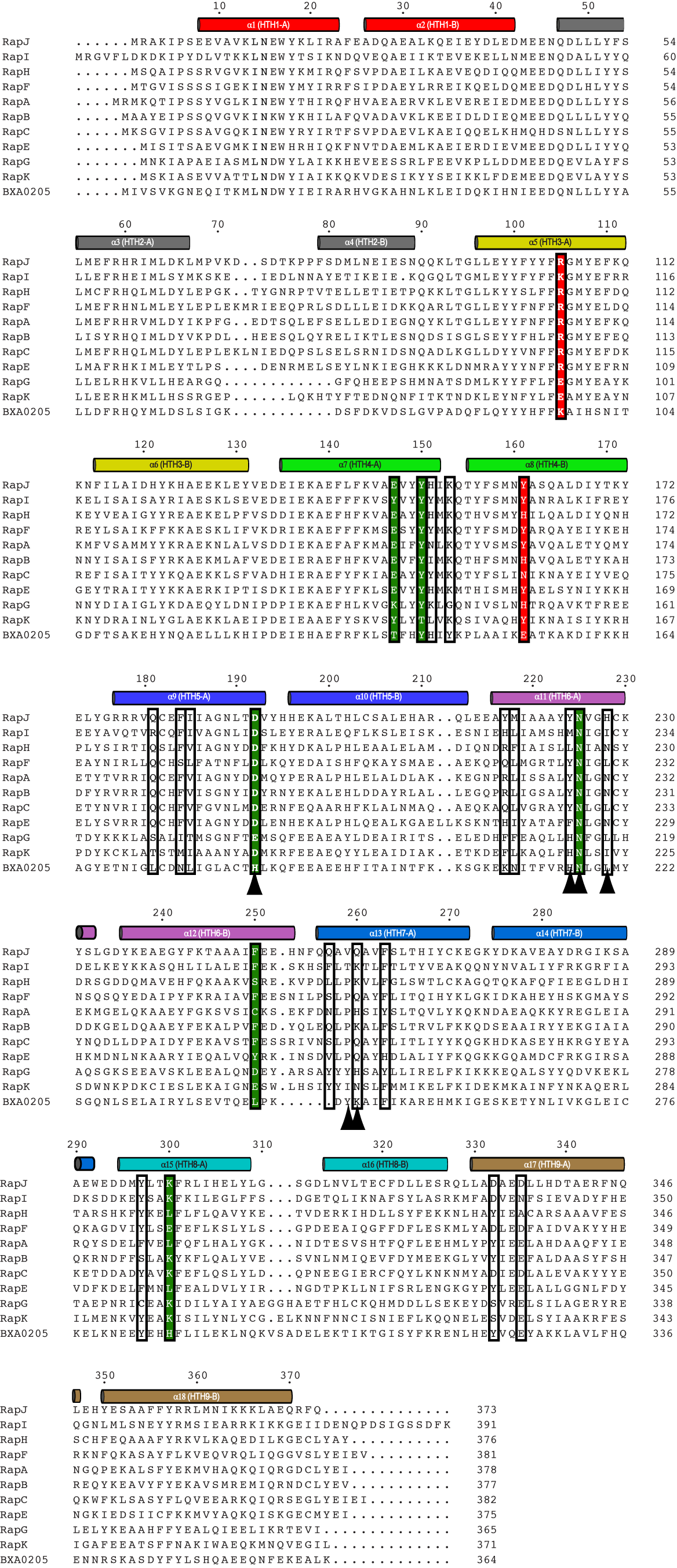

Supplement: Figure S5 — Rap protein sequence alignment. The amino acid sequences of Bacillus Rap proteins previously demonstrated to be regulated by Phr peptides were aligned using the MultipleAlignerClustalW method in STRAP [61]. The residue numbers indicated above the sequences refer to RapJ. The residues in the RapJ-PhrC interface are surrounded by black boxes. Residues marked with black arrowheads were previously shown to render RapA and RapC insensitive to PhrA and PhrC, respectively [10],[19],[24],[36]. Substitutions at the RapJ-PhrC interface residues highlighted by green boxes resulted in a complete loss of sensitivity to PhrC in vivo (Figure 6B). The highly conserved residue RapJ Asp192, which is highlighted by a green box and in bold type, makes a salt bridge with PhrC Arg2. Substitution mutations at residues participating in PhrC-driven RapJ intramolecular contacts (highlighted by red boxes) resulted in a severe loss of sensitivity to PhrC (Figure 7B). Similarly, RapH-R105A was insensitive to PhrH (Figure 7C). The colors of the cylinders representing α-helices correspond to the coloring scheme used in Figures 4, 5, and 7. (TIF) [file pbio.1001512.s005.tif]

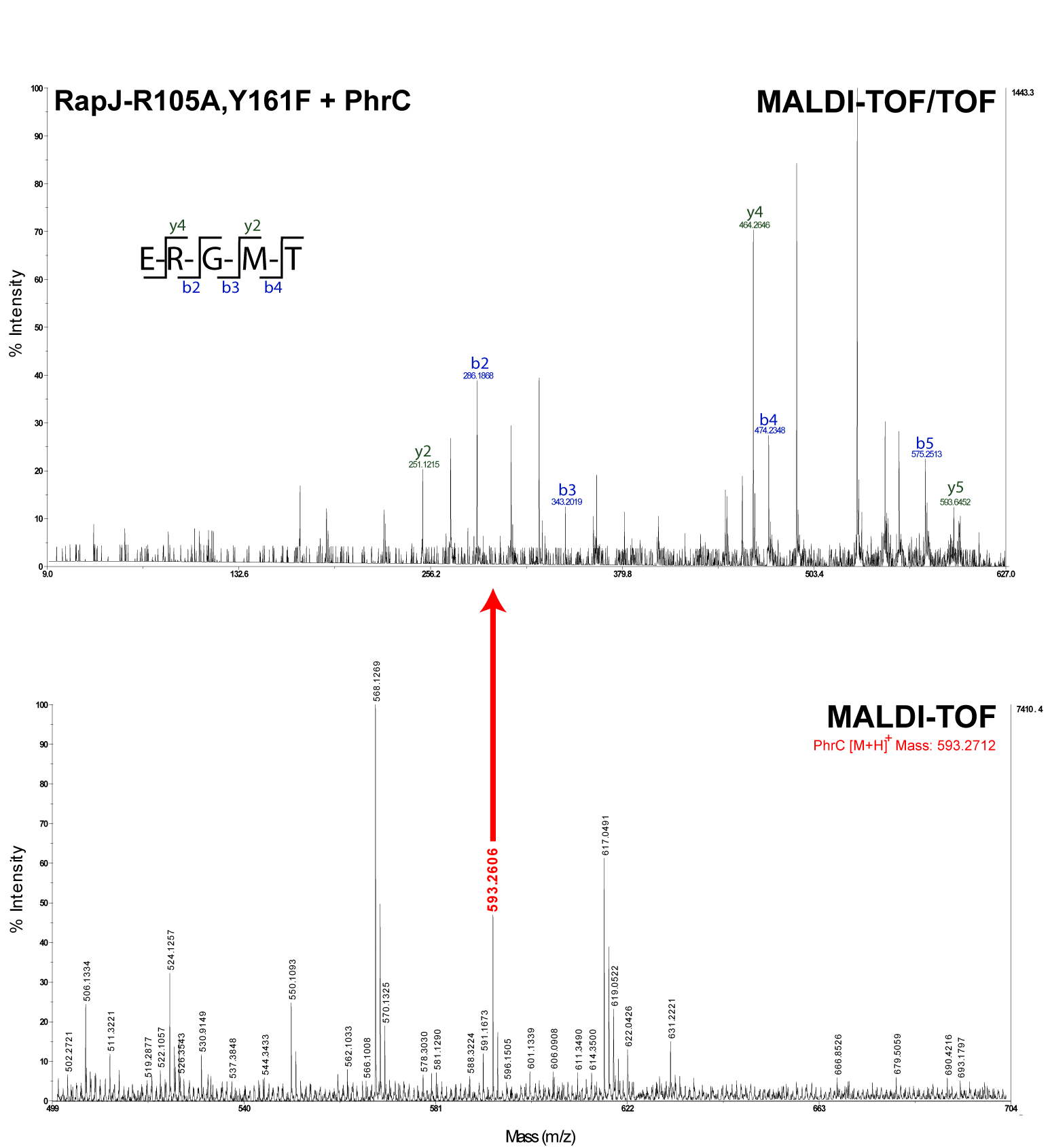

Supplement: Figure S6 — MALDI-TOF and MALDI-TOF/TOF tandem mass spectrometry of SEC purified RapJ-R105A,Y161F incubated with PhrC. (TIF) [file pbio.1001512.s006.tif]
