# Supplementary material for: Conformational Change-Induced Repeat Domain Expansion Regulates Rap Phosphatase Quorum-Sensing Signal Receptors
Source: PLoS Biol. 2013 Mar 19;11(3):e1001512. doi: 10.1371/journal.pbio.1001512 (PMC3601965; doi:10.1371/journal.pbio.1001512)
Supplement: Table S1 — Data collection and refinement statistics. Rsym = Σh Σi | Ii(h)−<I(h)>|/Σh Σi Ii(h), where Ii(h) is the ith measurement of h and <I(h)> is the mean of all measurements of I(h) for reflection h. Rwork = Σ ∥Fo|−|Fc∥/Σ |Fo|, calculated with a working set of reflections. Rfree is Rwork calculated with only the test set of reflections. Data for the highest resolution shell are given in parentheses. The structures were determined using single crystals. The reflections I(+) and I(−), related by Friedel's Law, were treated as independent for the purpose of the SAD data only. (DOC) [file pbio.1001512.s008.doc]

|  | **Native** | **SeMet SAD** | **Native** |
| --- | --- | --- | --- |
|  | RapJ-PhrC | RapJ-PhrC | RapI |
| **Data collection** |  |  |  |
| Space group | P4_3_2_1_2 | P4_3_2_1_2 | P2_1_2_1_2 |
| Cell dimensions |  |  |  |
| a, b, c (Å) | 86.80, 86.80, 225.87 | 86.92, 86.92, 226.44 | 132.90, 141.21, 50.53 |
| α, β, γ (°) | 90.0, 90.0, 90.0 | 90.0, 90.0, 90.0 | 90.0, 90.0, 90.0 |
| Resolution (Å) | 50.00-2.16 (2.20-2.16) | 50.00-2.21 (2.25-2.21) | 50.00-2.44 (2.48-2.44) |
| Wavelength (Å) | 0.9793 | 0.9793 | 1.075 |
| Completeness (%) | 99.9 (100.0) | 99.5 (100.0) | 99.1 (100.0) |
| R_sym_ (%) | 9.2 (68.7) | 7.9 (65.0) | 4.9 (64.9) |
| Average I / σI | 20.51 (5.51) | 17.10 (3.58) | 22.15 (4.53) |
| Redundancy | 14.05 | 7.77 | 7.18 |
| Total reflections | 663,706 | 644,886 | 257,751 |
| Unique reflections | 47,237 | 83,021 | 35,915 |
|  |  |  |  |
| **SAD Phasing** |  |  |  |
| Figure of merit |  | 0.35 |  |
|  |  |  |  |
| **Refinement** |  |  |  |
| R_work_ / R_free_ (%) | 18.97 (21.06)/22.51 (26.07) |  | 22.12 (26.83)/27.00 (31.42) |
|  |  |  |  |
| **Number of atoms** |  |  |  |
| All atoms | 6406 |  | 5365 |
| Protein | 6047 |  | 5337 |
| Oligopeptide | 80 |  |  |
| Ligand/ion | 2 |  | 2 |
| Water | 277 |  | 26 |
| **Average B-factor (Å^2^)** |  |  |  |
| All atoms | 47.08 |  | 76.44 |
| Protein | 47.50 |  | 76.48 |
| Oligopeptide | 30.38 |  |  |
| Ion | 49.21 |  | 91.03 |
| Water | 42.72 |  | 67.95 |
| **R.m.s. deviations** |  |  |  |
| Bond lengths (Å) | 0.002 |  | 0.007 |
| Bond angles (°) | 0.480 |  | 0.888 |
| **Ramachandran statistics** |  |  |  |
| Favored (%) | 99.58 |  | 97.77 |
| Allowed (%) | 0.42 |  | 1.75 |
| Outliers (%) | 0 |  | 0.48 |
|  |  |  |  |
