# Supplementary material for: Conformational Change-Induced Repeat Domain Expansion Regulates Rap Phosphatase Quorum-Sensing Signal Receptors
Source: PLoS Biol. 2013 Mar 19;11(3):e1001512. doi: 10.1371/journal.pbio.1001512 (PMC3601965; doi:10.1371/journal.pbio.1001512)
Supplement: Table S2 — RapI amino acid identity in highly conserved positions lying in the RapH-Spo0F interface [22]. RapA, RapB, RapE, RapH, and RapJ sequences refer to B. subtilis Rap proteins. BXA0205 and BA3790 sequences refer to B. anthracis Rap proteins. Sequences were aligned in Geneious Pro and analyzed using the ConSurf [63] server as previously described [22]. (DOC) [file pbio.1001512.s009.doc]

| RapH | RapA, RapB, RapE, RapJ, BXA0205, BA3790 | RapI |
| --- | --- | --- |
| I40 | I, I, I, L, I, I | L46 |
| E45 | E, E, E, E, E, Q | E51 |
| D46 | D, D, D, N, D, D | N52 |
| Q47 | Q, Q, Q, Q, Q, Q | Q53 |
| D48 | D, L, D, D, N, N | D54 |
| L50 | L, L, L, L, L, L | L56 |
| I51 | L, L, I, L, L, L | L57 |
| Y53 | Y, Y, Y, F, Y, Y | Y59 |
| L55 | L, L, L, L, L, L | L61 |
| F58 | F, Y, F, F, F, F | F64 |
| D65 | D, D, E, D, D, D | S71 |
| L96 | L, L, M, L, F, F | M100 |
| V132 | V, V, I, V, I, I | V136 |
| S133 | S, E, S, E, P, P | E137 |
| D134 | D, D, D, D, D, D | D138 |
| I136 | I, I, I, I, I, I | I140 |
| E137 | E, E, E, E, E, E | E141 |
| Y175 | Y, Y, Y, Y, Y, Y | Y179 |
