# Supplementary material for: Conformational Change-Induced Repeat Domain Expansion Regulates Rap Phosphatase Quorum-Sensing Signal Receptors
Source: PLoS Biol. 2013 Mar 19;11(3):e1001512. doi: 10.1371/journal.pbio.1001512 (PMC3601965; doi:10.1371/journal.pbio.1001512)
Supplement: Table S3 — Oligonucleotides. (DOC) [file pbio.1001512.s010.doc]

| **Name** | **Sequence** |
| --- | --- |
| RapJ-Fwd | 5′-GAGAACAGATTGGTGGTATGAGAGCAAAGATTCCATCAG-3′ |
| RapJ-Rev | 5′-CAGTCACCCGGGCTCGAGCTATTGAAAACGCTGCTCGG-3′ |
| ImmA-pBB-NdeI-Fwd | 5’-GAAGGAGATATACATATGATAACAATTTATAC-3’ |
| ImmA-pBB-EcoRI_rev | 5’-TATCTAGAGCTCGAATTCTTAACCAAAAGCGCTTA-3’ |
| ImmR1_F_pCOLANcoI_Inf | 5’-AGGAGATATACCATGGGCATGAGCCTAGGCAAACG-3’ |
| ImmR1_R_pCOLANotI_Inf | 5’-AGCATTATGCGGCCGCTTACTCTTTCTTCTTTAAT -3’ |
| RapI_Fwd_Infusion | 5’-CAGAGAACAGATTGGTGGTATGCGGGGTGTTTTC-3’ |
| RapI_Rev_XhoI | 5’-GTCACCCGGGCTCGAGTTACTTAAAATCACTGC-3’ |
| ΔrapJ_5'_Inf_F | 5’-CGACGGCCAGTGAATTCGCTGAGTTTTTCAGGGTG -3’ |
| ΔrapJ_5'_Inf_R | 5’-CTATTGAAAACGCTGCTCAATCTTTGCTCTCATCG-3’ |
| ΔrapJ_3'_Inf_F | 5’-CGATGAGAGCAAAGATTGAGCAGCGTTTTCAATAG-3’ |
| ΔrapJ_3'_Inf_R | 5’-CATGCCTGCAGGTCGACAAGTCCCCAATTCCGTC-3’ |
| pHyspank_rapJ_F | 5’-GAATTAGCTTGCATGCATGAGAGCAAAGATTCC-3’ |
| pHyspank_rapJ_R | 5’-ATTAAGCTTAGTCGACTTATTGAAAACGCTGCTC-3’ |
| rapJ_R105A_ChangeIT | 5’-TACTTTTATTATTTCGCAGGAATGTACGAATTC-3’ |
| rapJ_Y161F_Top | 5’-GTATTTTTCCATGAATTTTGCGAGCCAGGCGCTT-3’ |
| rapJ_Y161F_Bottom | 5’-AAGCGCCTGGCTCGCAAAATTCATGGAAAAATAC-3’ |
| rapJ_K300E_ChangeIT | 5’-GACATGTACTTGACGGAATTCCGCCTCATTCATGA-3’ |
| rapJ_D192A_ChangeIT | 5’-CGCCGGAAATTTGACCGCTGTGTATCATCATGAAA-3’ |
| rapJ_Y150F_ChangeIT | 5’-GTCGCAGAAGTGTATTTTCACATCAAACAAACGT-3’ |
| rapJ_N225A_ChangeIT | 5’-CGCTGCTGCCTATTATGCTGTCGGACACTGTAAGT-3’ |
| rapJ_E147A_ChangeIT | 5’-CTTTTTAAAGTCGCAGCAGTGTATTATCACATCA-3’ |
| rapJ_F250A_ChangeIT | 5’-ACAGCCGCCGCCATTTGTGAGGAGCACAACTTTC-3’ |
| rapJ_E87A_ChangeIT | 5’-ATGCTGAACGAAATTGCAAGCAATCAGCAAAA-3’ |
